# Quality by design modelling to support rapid RNA vaccine production against emerging infectious diseases

**DOI:** 10.1038/s41541-021-00322-7

**Published:** 2021-04-29

**Authors:** Damien van de Berg, Zoltán Kis, Carl Fredrik Behmer, Karnyart Samnuan, Anna K. Blakney, Cleo Kontoravdi, Robin Shattock, Nilay Shah

**Affiliations:** 1 Centre for Process Systems Engineering, Department of Chemical Engineering, Faculty of Engineering, Imperial College London, London, UK; 2 Department of Infectious Disease, Faculty of Medicine, Imperial College London, London, UK; 3 Present Address: University of British Columbia, Michael Smith Laboratories and School of Biomedical Engineering, Vancouver, BC Canada

**Keywords:** RNA vaccines, RNA vaccines, Drug development

## Abstract

Rapid-response vaccine production platform technologies, including RNA vaccines, are being developed to combat viral epidemics and pandemics. A key enabler of rapid response is having quality-oriented disease-agnostic manufacturing protocols ready *ahead* of outbreaks. We are the first to apply the Quality by Design (QbD) framework to enhance rapid-response RNA vaccine manufacturing against known and future viral pathogens. This QbD framework aims to support the development and consistent production of safe and efficacious RNA vaccines, integrating a novel qualitative methodology and a quantitative bioprocess model. The qualitative methodology identifies and assesses the direction, magnitude and shape of the impact of critical process parameters (CPPs) on critical quality attributes (CQAs). The mechanistic bioprocess model quantifies and maps the effect of four CPPs on the CQA of effective yield of RNA drug substance. Consequently, the first design space of an RNA vaccine synthesis bioreactor is obtained. The cost-yield optimization together with the probabilistic design space contribute towards automation of rapid-response, high-quality RNA vaccine production.

## Introduction

The outbreak and spread of viral diseases, such as the COVID-19 pandemic caused by the SARS-CoV-2 virus, the 2015–2016 Zika virus epidemic in Brazil and American continents, the re-emerging Nipah outbreaks in South and Southeast Asia, and the 2013–2016 Ebola virus epidemic in West Africa, pose tremendous healthcare and economic challenges^1–3^. Vaccines are highly effective for stopping epidemics and pandemics. However, the development of vaccines using conventional production methods is becoming too slow to effectively respond to new viral outbreaks in the 21st century^4^, the frequency of which is predicted to increase^3^.

To address this pressing need, rapid-response vaccine production platform technologies are being deployed, such as the messenger RNA (mRNA) and self-amplifying RNA (saRNA) platforms, herein collectively referred to as RNA vaccine platforms. The mRNA and saRNA vaccine production process involves cell-free DNA-templated RNA synthesis based on the in vitro transcription (IVT) reaction catalysed by the T7 RNA polymerase enzyme (T7RNAP)^5,6^. The RNA (both mRNA and saRNA) drug substance is purified using tangential flow filtration (TFF) and chromatography techniques, such as ion-exchange or multimodal chromatography^4,7^. Then the RNA drug substance is formulated into lipid nanoparticles and filled into vials or other containers^4,7^. A process diagram showing RNA vaccine drug substance and drug product manufacturing are shown in Supplementary Fig. 1.

RNA vaccines involve rapid development and production timelines because the production platform is agnostic to the disease target as RNA sequences translating into any vaccine protein antigen can be produced using the same production process^8^. The only component in this production process that needs to be changed is the template DNA based on which the RNA is enzymatically synthesised. The rest of the materials, equipment, consumables, unit operations, formulation components, fill-to-finish processes as well as quality control and quality assurance methods remain unchanged when switching to the production of a new RNA sequence encoding for a new vaccine antigen. This is possible because the RNA vaccine manufacturing process produces only the genetic instructions for expressing an antigen in human cells, and not the actual antigen. Using this technology, candidate vaccines can be produced against any known or currently unknown future pathogens. For example, mRNA and saRNA vaccine candidates against COVID-19 have been recently produced with an unprecedented speed: in 2 weeks after obtaining the genetic sequence information of the antigen^9,10^. The mRNA vaccines developed by BioNTech and Moderna gained emergency use authorisation against Covid-19 at record speed, despite the RNA vaccine platform being a new technology that had not been approved by regulatory authorities in the past.

The development of process monitoring and quality assurance approaches remains a key challenge for quickly and cost-effectively ensuring that the drug substance is produced with consistently high quality. This should be explored and developed *prior* to the production of a particular product, ideally in a disease- and product-agnostic manner to complement the flexible manufacturing platform of vaccine candidates against a wide range of pathogens. The quality by design (QbD) framework has been used to aid the regulatory approval and production of small molecule pharmaceuticals^11,12^ and monoclonal antibodies^13,14^ by establishing a design space (DS) in which the production process can be operated to consistently obtain the required quality target product profile. Regarding vaccines, some are currently in development based on QbD frameworks^15,16^. However, to the knowledge of the authors, there are currently no vaccines approved by regulatory authorities based on a full QbD filing. The QbD framework consists of two key steps: (1) a risk assessment based on the identification of product critical quality attributes (CQAs) and critical process parameters (CPPs) and (2) definition of the DS in the CPPs space which is obtained by defining mathematical relationships between CPPs and CQAs. For the first step, quality attributes are commonly ranked based on their impact and uncertainty scores for both product safety and efficacy, obtaining this way a severity score based on which the CQAs are identified^15^. Next, the CPPs are identified by assessing the impact of PP ranges on the identified CQAs, predominantly by using a binary, yes or no, approach based on expert knowledge and product-process understanding^15^. Alternatively, CQAs and CPPs can also be identified and ranked using fishbone diagrams, cause–effect matrices and failure mode effect analyses^17–19^. However, none of these existing methods is able to capture the direction, magnitude and shape of the impact of the CPPs on the CQAs. Therefore, more advanced approaches are needed to better describe the relationship between CPPs and CQAs in a data-poor environment, which is typical to the early phases of production process development.

To address this need, we developed and implemented a new qualitative QbD methodology that assesses the criticality of PPs considering the direction, magnitude and shape of the CPP–CQA relation. Furthermore, we developed a bioprocess model to map the multi-dimensional DS of RNA synthesis substantially faster and with fewer resources compared to the experimental design of experiments (DoE) protocols. This bioprocess model was built on previously published RNA synthesis kinetics^20^. Such mechanistic models tend to outperform statistical or data-driven (e.g. machine learning) models in data-poor environments, such as during the early stages of process development. This is the first bioprocess model of an RNA vaccine synthesis bioreactor in support of DS identification and optimisation. The proposed qualitative QbD methodology which maps the direction, magnitude and shape of the impact CPP–CQA relation together with the bioprocess model forms the QbD framework. Overall, the framework is to become universally applicable to mRNA and saRNA vaccine manufacturing using wild-type nucleotide triphosphate NTPs and is *independent* of the viral infectious disease indication, because both the RNA vaccine manufacturing process and the QbD framework can be applied to produce any antigen-encoding RNA sequence^4,5^. The QbD framework applied to the RNA platform further supports upstream process optimisation during both development and manufacturing and is anticipated to expedite the regulatory approval process by providing a form of “pre-qualification” by re-using and processing disease agnostic-prior knowledge^4^.

## Results

### QbD framework

**Fig. 1:**
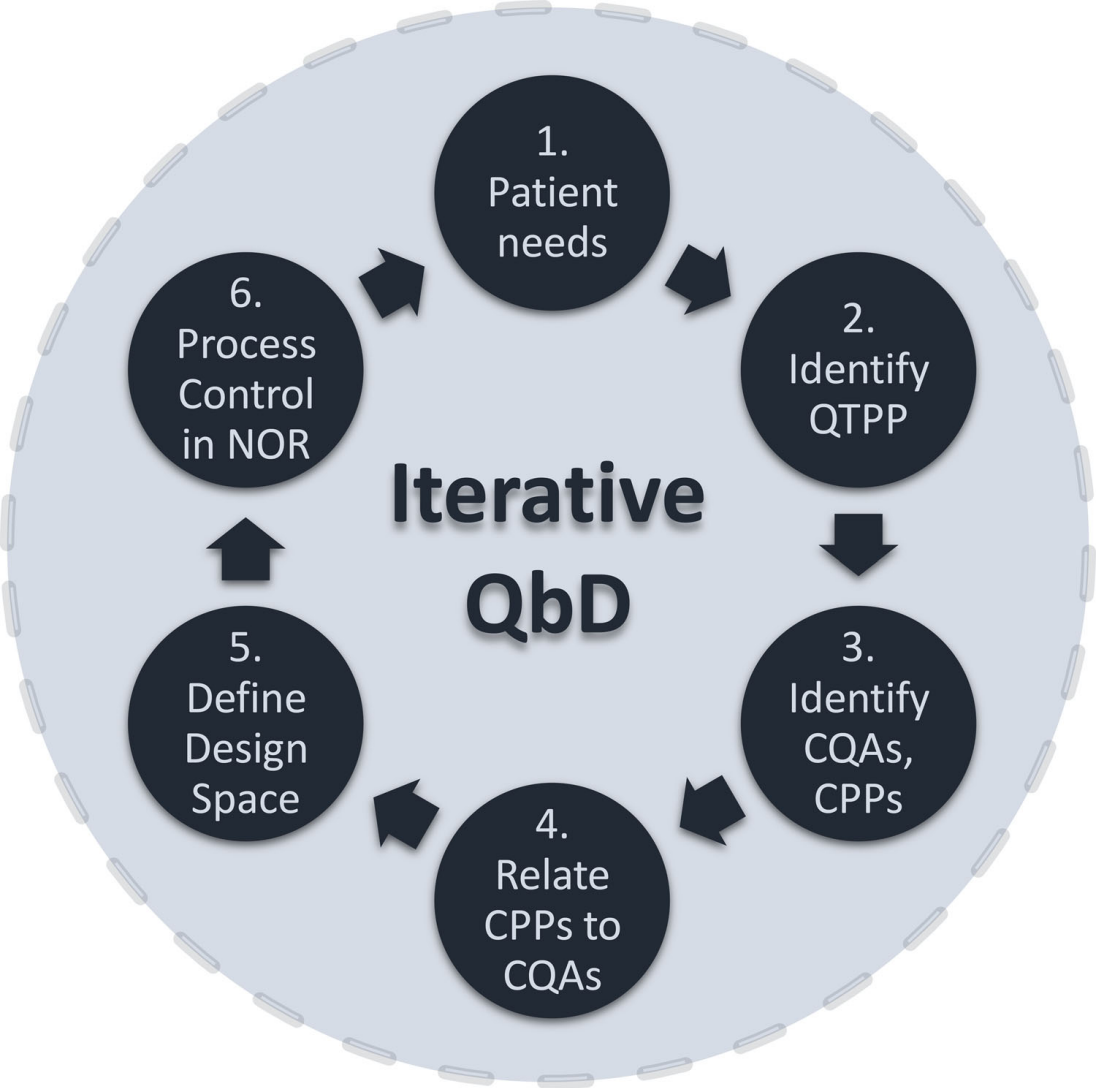
Quality‐by‐design (QbD) framework development cycle. The QbD development cycle starts with identifying the needs of the patients and from there the Quality Target Product Profile (QTPP) is determined. Based on the QTPP, the critical quality attributes (CQAs) of the product and their ranges are determined using a risk assessment scoring^15^, taking into account clinical and non‐clinical data, for both product safety and efficacy. Next, based on understanding how the production process unit operations impact the product CQAs, the critical process parameter (CPP) ranges are defined. From this product-process understanding, mathematical relations between CPPs and CQAs are established, thus obtaining a mathematical model of the vaccine production process at the unit operation level. This model is then used to identify the ranges of CPPs which yield the desired CQAs. From these CPP ranges, the design space can be created and therein a sub-space termed the normal operating range (NOR) is defined. The production process can be operated in NOR also by adapting the QbD bioprocess model for advanced process control, using model predictive control which takes in real‐time measurement data from the production process. The “digital twin” based automation in the NOR allows for real-time optimisation of the production process which follows current good manufacturing practices (cGMP)^49,50^; manufacturing products for the patients’ needs at consistently high quality. Thus, the QbD framework supports both the development and operation of production processes and it follows an iterative development cycle to ensure continuous improvement through the product‐process life cycle^4,15^.

The mRNA and saRNA and their intrinsic quality features are created during the in vitro transcription (IVT) reaction, therefore the QbD framework, consisting of a qualitative methodology and a quantitative bioprocess model, has been applied to this unit operation. As shown in Fig. 1, the QbD framework development cycle starts with patient need identification and quality target product profile definition. This is followed by CQA and CPP definition, CQA–CPP relation, DS and normal operating range (NOR) definition and, finally, production process automation and control using model predictive control (digital twins).

### CQAs and CPP identification

In the third step, the qualitative methodology is used to identify and rank the CQAs of the mRNA and saRNA vaccine, as shown in Fig. 1. The listing and ranking of CQAs are shown in Supplementary Table 1. The four CQAs identified were: RNA yield, sequence integrity, sequence identity and 5′ capping efficiency. CPP identification and CPP–CQA relation were then established using a novel qualitative ranking methodology, as shown in Table 1. This considers the direction, magnitude and shape of the impact of the CPPs on the CQAs, as described in the “Methods” section.

**Table 1:** Proposed qualitative framework for the assessment of the criticality of process parameters (PPs) based on the qualitative assigned impact of PPs on the CQAs^a^.

Process parameter	RNA sequence integrity	RNA sequence identity	RNA yield	Source^b^	Classification
Temperature	−2	−1	±3	Data, Expert^51^	CPP
Pressure	±1	±1	±1	Expert	PP
Mixing	±1	±1	±1	Expert	PP
Reactor dimensions	0	0	0	Expert	PP
Reaction time	±2	0	3	Data, Expert	CPP
pH in transcription reactor	±2	±2	±3	Data, Expert^52,53^	CPP
DNA template sequence	−2	−3	0	Expert	CPP
DNA template concentration	0	0	+2	Data, Expert	CPP
T7RNAP concentration	±1	±1	+3	Data, Expert	CPP
5’ cap analogue concentration	0	±3	0	Data, Expert	CPP
Total Mg concentration	0	±2	±3	Data, Expert^44^	CPP
DTT concentration	±2	±2	±1	Data, Expert^45^	CPP
Spermidine concentration	±2	±2	±3	Data, Expert^46^	CPP
GTP concentration	+1	±3	±3	Data, Expert	CPP
Total NTP concentration	+2	±2	±3	Data, Expert	CPP
Ratio of NTPs	+1	±3	±3	Expert	CPP

The PP-CQA relationship is characterised by an impact magnitude rating, a sign indicating directionality and shape of the CQA = *f* (PP) plot. The magnitude of the impact was rated from 0 (low) to 3 (high), and PPs rated with 2 or 3 were considered critical, thus critical process parameters (CPPs). The direction and type of CPP-CQA relationship were characterised either by a positive slope labelled with plus “+”, a negative slope labelled with a minus “−”, or a peak behaviour whereby the CQA increases with increasing the PP reaches a peak and then decreases, labelled with plus-minus “±”.

^a^The CQAs of “Bacterial endotoxins”, “Bioburden”, 5’ capping efficiency and “Post-filtration pH” from Supplementary Table 1 were not included in this table because these can be assumed to be well-controlled in a GMP bioproduction process.

^b^The ratings provided in the “RNA sequence integrity”, “RNA sequence identity” and “RNA Yield” columns were based on experimental data (Data), information from the literature (where applicable reference is included and are listed in the main article bibliography) and expert knowledge (Expert).

### Bioprocess model development

The four CQAs from Supplementary Table 1 were grouped into one output, termed effective RNA yield. The 5′ capping efficiency CQA was not modelled individually because the commercially available 5′ cap analogue, CleanCap (TriLink Biotechnologies, San Diego, CA, USA) yields 5′ capping efficiencies of ≈95% which is sufficient for the expression of the vaccine antigen from the RNA transcript in human cells^21–25^. The bioprocess model involves bi-substrate kinetic formulae for the transcription reaction, adapted from a previously published multiphysics kinetic model^20^, to compute the RNA transcription yield. Given the prior knowledge that RNA degrades in alkaline as well as acidic environments^26^, and that high Mg^2+^ concentration favours RNA degradation^27^, RNA degradation rate was modelled as a series of three power laws, each first order in RNA and first-order in either proton, hydroxy or Mg^2+^ concentration. Four CPPs identified in Table 1 were included in the model: initial total solution wild-type nucleotide triphosphate (NTP) and Mg concentrations, T7RNAP concentration, and reaction time. There is a clear distinction in notation between the use of Mg^2+^ and Mg. Mg^2+^ is used to refer to free solution magnesium, while Mg is used when referring to total magnesium concentration in free solution together with magnesium in complexes, often in the context of initial experiment conditions. The remaining nine CPPs were not considered in this RNA synthesis bioreactor model because these CPPs can be well controlled in commercially available bioreactor setups implemented in facilities following cGMP guidelines.

**Fig. 2:**
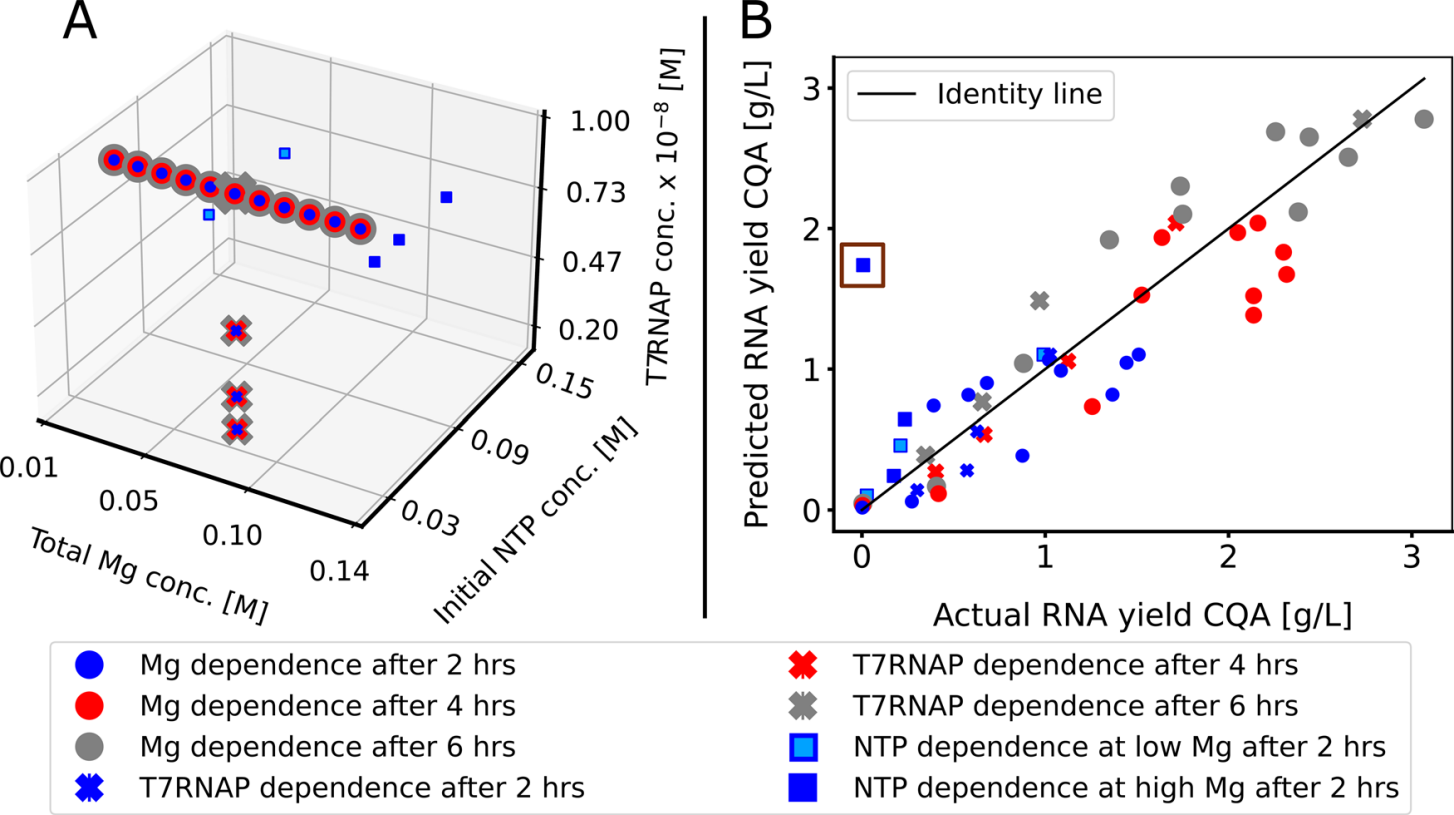
Experimental data distribution and modelling error plots. **A** Three-dimensional plot showing the experimental data generated using a statistical Design of Experiments approach. Data points of different colour overlap. This data was used for model calibration and validation. **B** Modelling error plot. The *x*-axis represents the true experimental RNA yield, and the *y*-axis marks the corresponding prediction from the model. Each point (circle, square or *x*) is a prediction generated using the model. The black line represents the identity line, where modelling results perfectly match the experimental outcome. The brown square encases the outlier for NTP dependence at high Mg concentration. The meaning of the colours is indicated in the legend below the plots.

The model parameters were then fitted to a subset of 51 experimental samples from a statistical DoE dataset obtained from lab-scale saRNA synthesis experiments using wild-type NTPs^28^. This dataset includes NTP and T7RNAP screening experiments, and more thorough analysis of RNA yield surface response on Mg concentration. 33 samples correspond to the RNA yield at 0.04 M NTP and 1 × 10^−8^ M of T7RNAP vs. 11 concentrations of Mg ranging from 0.025 to 0.125 M after 2, 4 and 6 h (circles in Fig. 2). Twelve samples correspond to the RNA yield at 0.04 M NTP and 0.075 M Mg for 1.250 × 10^−9^, 2.5 × 10^−9^, 5 × 10^−9^ and 1 × 10^−8^ M of T7RNAP after 2, 4 and 6 h (crosses in Fig. 2) and 6 samples correspond to the RNA yield after 2 h at 0.02, 0.04 and 0.08 M NTP at 0.075 and 0.14 M Mg (squares in Fig. 2). For additional information about the experimental data see^28^. The kinetic equations describing the RNA yield response correspond to Eqs. (1)–(10). The parameter estimation found *k*_app_ to be 4.34 $\frac{{\rm{L}}^2}{{\rm{mol}}\,{\rm{U}}\,{\rm{h}}}$, *K*_1_ 5.55 × 10^5^ $\frac{\rm{L}}{\rm{mol}}$, *K*_2_ 1.94 × 10^5^ $\frac{\rm{L}}{\rm{mol}}$ and *k*_ac_ 1.20 × 10^6^ $\frac{\rm{L}}{{\rm{mol}}\,{\rm{h}}}$, while the effect of *k*_ba_ and *k*_Mg_ was found to be negligible. It has to be noted that multiple parameters such as *k*_app_, *K*_1_ and *K*_2_ were highly correlated, meaning multiple combinations thereof gave the same dynamic response.

To mitigate this co-correlation, insignificant parameters could be fixed. To this effect, a variance-based global sensitivity analysis was performed around the optimal parameter values as determined by the parameter estimation^29–33^. This analysis helps to evaluate how uncertainty propagates from the kinetic model parameters to the RNA yield and to quantify how much of the variation in the RNA yield can be attributed to the individual kinetic model parameters^29–33^. As expected, *k*_ba_ and *k*_Mg_ were found to be negligible, with Sobol indices below 0.001, thus contributing less than 0.1% to the variation in RNA yield computed by the model after 6 h of IVT reaction time, cf. Supplementary Table 3 in the SI document. On the other hand, *k*_app_ was found to be most significant as the only parameter driving the reaction forward, explaining over 60% of the model-predicted RNA yield variation after 6 h of IVT reaction time, cf. Supplementary Table 3 in the SI document. Higher values of these Sobol indices, which are ANOVA-decomposed variance contributions, indicate stronger dependence of the variation in the RNA yield on the respective kinetic model parameters^29–33^. Thus, the significance of parameters can be ranked in this decreasing order *k*_app_, *K*_1_, *K*_2_, *k*_ac_ with Sobol indices of 0.61, 0.30, 0.06 and 0.03, respectively. *k*_app_ and *K*_1_ together explain over 91% variation in the RNA yield after 6 h of IVT reaction time. The Sobol index table as well as the scatter plots of RNA yield after 6 h plotted in function of kinetic model parameters can be found in Supplementary Table 3 and Supplementary Fig. 2.

Model fit to experimental data were generally good, capturing most of the non-linearities and overall trends with no consistent over- or underestimation bias, as seen in the prediction error plot in Fig. 2. The modelling mean absolute error (MAE) of 0.28 g/L is acceptable given that the standard deviation in experimental data samples can be as high as 0.95 g/L. The average prediction error is expected to decrease in future QbD framework iterations as more data and knowledge become available. However, there is one striking outlier predicting RNA yield to be high at both high Mg and NTP concentrations, while it should be close to zero. This outlier sample, as shown in Fig. 2, gives the RNA yield to be 0.01 g/L after 2 h at starting conditions of 0.14 M Mg, 0.08 M NTP and 1 × 10^−8^ M T7RNAP, corresponding to the encased dark blue square in Fig. 2. With the Mg concentration fixed at a high 0.14 M, in its current iteration, the model captures the increase in RNA yield from 0.02 to 0.04 M NTP but not the subsequent decrease thereof from 0.04 to 0.08 M NTP, after which the rate of RNA production should be almost zero. The failure of the model to predict this sample point can be explained by model overfitting to the many samples describing Mg dependence compared to the few samples at different NTP concentrations. The addition of other model terms could support a more accurate prediction at high Mg and NTP concentrations but would lead to even worse testing performance through over-parameterisation.

On top of the dataset being skewed towards the dependence on Mg and T7RNAP rather than NTP, many of the RNA yield values are clustered close to zero, c.f. Supplementary Table 4 for the descriptive statistics on the RNA yield dataset. Before performing a quantitative model-based DoE, qualitative suggestions for further experiments can be proposed to increase the statistical significance of the model and to more accurately account for the peak in RNA yield at increasing NTP concentrations. The following experiments would lead to a smoother regression around the experimentally optimal region: measure the RNA yield at each Mg concentration of 0.06, 0.075 and 0.090 M for NTP concentrations of 0.02, 0.03, 0.05 and 0.06 M after 2, 4 and 6 h of IVT reaction time. More useful still might be the inclusion of physical variable measurements other than RNA yield. These could include free solution NTP^4−^ concentration (if analytically distinguishable from NTP in the transcribed RNA chain), solution turbidity due to Mg_2_PPi precipitating after the formation of PPi as a byproduct and pH. Through such measurements, one can more easily discriminate the relative importance of the physical phenomena contributing to the degradation of RNA. Ultimately, these measurements would also help in determining tighter bounds on *k*_app_ as the most significant parameter.

Despite these current limitations, the mechanistic model performs well in comparison to conventional statistical modelling techniques. Multiple linear regression (MLR) using four linear explanatory variables (four coefficients plus a constant) gave an *R*^2^ value of 0.398 and an MAE of 0.570 g/L due to its inability to capture non-linearities. Only after including squared terms in both Mg and NTP and their interaction term in the regression (seven coefficients plus a constant), did the fit of the statistical model increase to an *R*^2^ value of 0.766 and an MAE of 0.167 g/L, which are comparable to that of the mechanistic model (0.773 and 0.162 g/L, respectively). The summary of the models’ prediction plots and descriptive statistics can be found in Supplementary Fig. 3 and Supplementary Table 4 to Supplementary Table 6.

### Model implementation and DS definition

The current model performed well in the region of interest at medium-to-low Mg and NTP concentrations and hence this model was used to create the first DSs. In conjunction with cost and safety considerations, this leads to first recommendations about the desired operating region and subsequent experimental design. The limitations of the model at high Mg and NTP concentrations will be resolved in future iterations with the use of additional experimental data that will be obtained from optimal experiment designs.

**Fig. 3:**
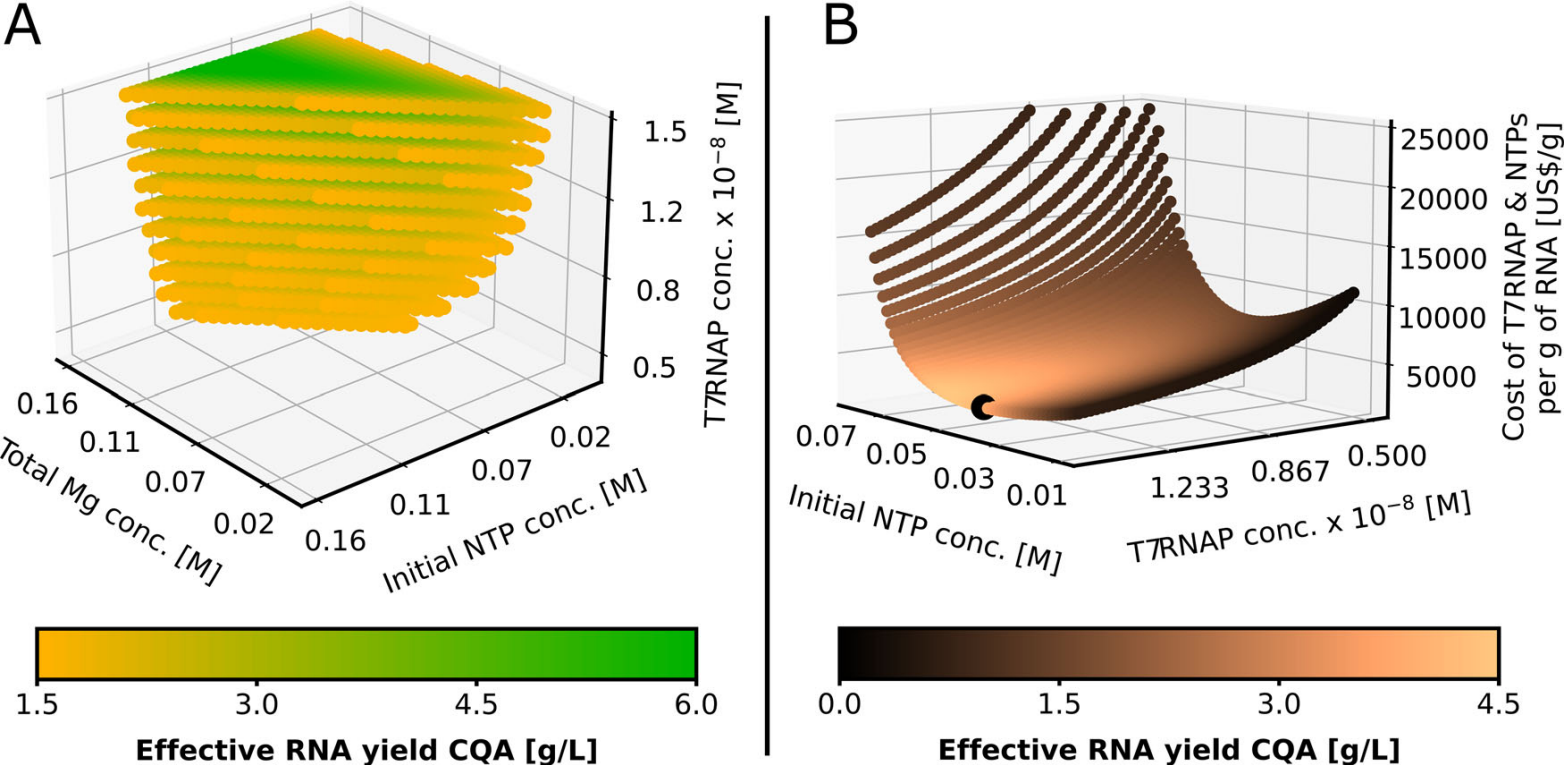
Deterministic design space (DS) and cost per yield surface. **A** Three-dimensional deterministic DS mapping the relationship between CPPs initial total solution Mg, NTP and T7RNAP concentration with the effective RNA yield CQA. Every point within the DS meets the 1.5 g/L RNA production target and the greener the region, the higher the yield. **B** Yield-cost-concentration plot showing RNA production yield and T7RNAP and NTP cost per g of RNA in the function of T7RNAP and NTP concentrations at 85 mM constant Mg concentration. The black sphere marks the cost optimum.

The deterministic DS and concentration-cost-yield plots produced by the model are shown in Fig. 3. Figure 3A produces the deterministic DS after 6 h defined by the remaining three CPPs, with the optimum corresponding to high RNA effective yield shown by the green region. At fixed T7RNAP, the DS also shows that at fixed initial Mg or NTP, the concentration of the other component passes through an optimum. The optimum in Mg at fixed NTP can be seen more clearly at low NTP.

In addition to obtaining high values for the RNA effective yield, the product should also be produced at low cost. For this, Fig. 3B shows the yield-cost-concentration plot, whereby the costs of the T7RNAP and NTPs were optimised per g of RNA and the other production costs components were assumed fixed and were not part of the cost optimisation objective. Figure 3B indicates a positive, linear correlation between the T7RNAP concentration and RNA yield. The T7RNAP concentration appears as a first-order reactant in the modelled transcription reaction and does not contribute to the degradation of transcribed RNA. However, increasing T7RNAP concentration incurs higher cost. Thus, costs of T7RNAP and NTPs expressed per g of RNA are shown on the *z*-axis of Fig. 3B and the yield is indicated by the colour map. Initial Mg concentration is fixed as its cost contributes only a negligible amount compared to T7RNAP and NTPs. A fixed initial concentration of 85 mM for Mg was chosen as this corresponded to the experimental optimum. The minimum of 2740 $ costs of T7RNAP and NTPs expressed per g of RNA is shown by the black sphere at 1.5 × 10^−8^ M T7RNAP concentration, at 40.8 mM NTP concentration and at an RNA yield of 4.34 g/L, as shown below in Table 2.

**Table 2:** Key modelling input and output results.

I/O	Parameter	Unit	Value
Input	NTP concentration	mM	40.8
	T7RNAP concentration^a^	M	1.5 × 10^−8^
	Mg concentration^b^	mM	85
Output	Yield in bioreactor	g × L^−1^	4.34
	Cost of T7RNAP and NTPs per g of RNA	USD × g^−1^	2740

^a^The T7RNAP concentration range for optimisation was 0.5 × 10^−8^−1.5 × 10^−8^.

^b^The Mg concentration was the experimental optimum and it was not subject to cost-yield optimisation due to the low purchase cost of this material.

The results indicate that the relatively high NTP concentration contributes more to the RNA cost per gram compared to the lower concentration of T7RNAP and that cost optimality is reached at the highest T7RNAP concentration. This holds true as long as RNA yield continues to grow linearly with T7RNAP, or as long as the solution is rich in NTP. The range of T7RNAP and NTP concentrations for which this is valid needs to be investigated through further experiments.

However, given that at the experimental optimums NTPs are relatively more expensive than the other two components, the operating point should be chosen at the minimum NTP concentration that ensures reaching the desired CQA with a certain probability. The cost values reported above correspond to the cost of the T7RNAP and NTPs expressed per g of RNA, but this is not the total production cost of the RNA drug substance. In fact, the major cost driver in RNA vaccine production is the 5′ cap analogue purchase price. The concentration of the 5′ cap analogue remains unchanged when RNA vaccines of different length are used due to the very high molar excess of the 5′ cap analogue used relative to the final molar RNA concentration; the calculations are available in Supplementary Table 2.

Production cost components other than the cost of the T7RNAP and NTPs were not included in this model as they were considered fixed. For a detailed analysis of the RNA vaccine drug substance production cost see^7^.

**Fig. 4:**
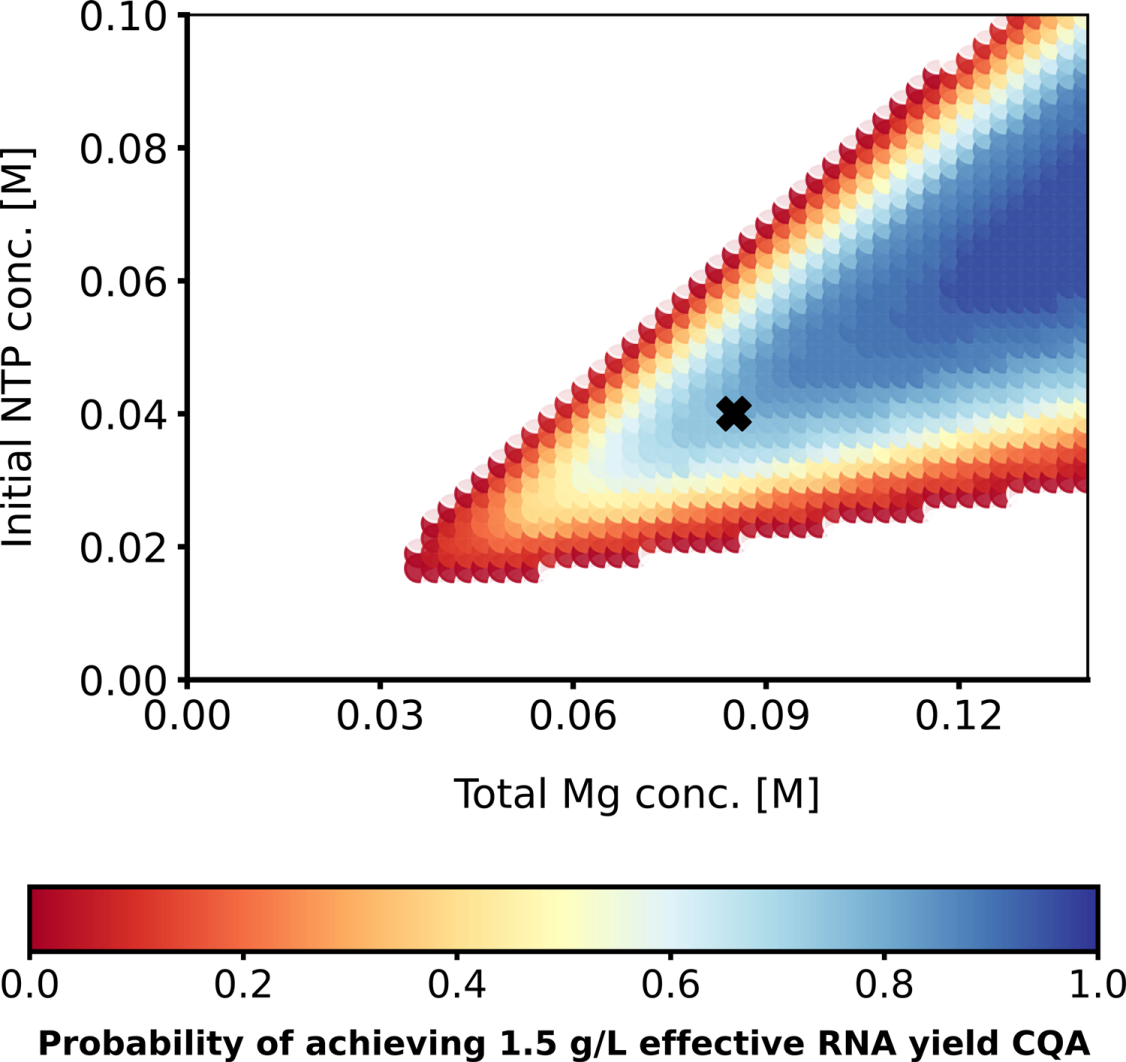
Two-dimensional probabilistic design space. The probability of achieving the 1.5 g/L RNA effective yield CQA under 20% standard deviation in the kinetic rate constant model parameters at a fixed 10^−8^ M T7RNAP concentration. The probability is illustrated by the colour code. The black cross represents the cost-optimal point.

To address uncertainties and ascertain process operational flexibility, a probabilistic DS was created by adding 20% standard deviation to the fitted model parameters from Eq. (3). Monte Carlo simulation results are shown at constant 1 × 10^−8^ M T7RNAP concentration in Fig. 4. The cost-optimal operating point is marked with a black cross and reaches the desired CQAs with a probability of 75–80%. Note that a high standard deviation of 20% was chosen to represent both model and process uncertainties. As more knowledge becomes available about the system, the uncertainty is reduced. In later iterations, the DS should not spread as far out to high Mg and NTP concentrations as the model prediction in this region was already shown to be poor due to the limited training dataset.

## Discussion

The emergency use authorisation granted to the BioNTech/Pfizer and Moderna mRNA vaccines underlines the crucial importance of this new vaccine platform technology, which succeeded in developing and producing vaccines against a new coronavirus at record speeds even though it was a new technology that had never gained regulatory approval in the past. The mRNA platform is also well-positioned to rapidly deploy vaccines against new SARS-CoV-2 variants. saRNA vaccines are also being developed^34,35^ offering additional benefits through increased production volumes and speeds and reduced production costs^4,7^. The QbD framework presented herein aids the acceleration of the development and manufacturing of both mRNA and saRNA vaccines, here collectively referred to as RNA vaccines.

The QbD framework is anticipated to expedite the regulatory approval process by providing a form of “pre-qualification”^4^. This “pre-qualification” is facilitated by the platform nature of both the RNA vaccine manufacturing process and of the QbD framework. The mRNA and saRNA vaccine production platforms facilitate this “pre-qualification” provision by re-using disease-agnostic prior knowledge, production process understanding, expert knowledge, experimental and clinical data from old RNA vaccines to produce new RNA vaccines and vaccine candidates. The QbD framework aids this “pre-qualification” by processing all the information from the mRNA and saRNA vaccine development and manufacturing processes to obtain the optimal outcomes in terms of product safety, efficacy and cost.

The QbD framework incorporates disease-agnostic prior knowledge, production process understanding, expert knowledge, current experimental and clinical data. This framework can serve as a “pre-qualification” for speeding up pre-clinical and clinical development and regulatory approval processes for future outbreaks. Such a framework is especially beneficial when combined with a vaccine production platform technology because both the RNA platform and the QbD framework are disease-agnostic. The implementation of the QbD framework follows an iterative development cycle, as shown in Fig. 1^4^. Within this, the criticality of product quality attributes and PPs is evaluated. Next, the impact of CPPs on CQAs is assessed using the qualitative methodology shown in Table 1. This streamlines the development of QbD models and the establishment of a DS. The DS presented here is the first published for an RNA vaccine production process.

The next step in the QbD framework would include defining the NOR within the DS as a safety margin against the process and material fluctuations, model uncertainties and other uncertainties. This allows for operational flexibility in a production process following current Good Manufacturing Practices (cGMP), offering substantial advantages compared to a conventional “frozen” cGMP process in which the operating parameters are fixed. The NOR can be defined from the probabilistic DS, shown in Fig. 4, and based on financial cost considerations, shown in Fig. 3B. Generating a surrogate model of the QbD model and adapting it for model-predictive control then enables advanced automation. To achieve this, for example, the model can predict undesired changes in product quality in the near future (e.g. in the next 5 min) and these predicted alterations in product quality will be linked to production PPs. Model-based control will be able to rapidly (e.g. within seconds) determine the corrective control actions that will lead to the optimal set of PPs which will counteract the predicted undesired changes in product quality. Thus, the model predictive controller will be able to correct the predicted faults in product quality before these would occur in the first place. This approach will ensure consistent product quality even under inherent process fluctuations while maximising effective RNA yield at the lowest possible cost.

The RNA platform combined with the QbD framework is suitable for producing vaccines rapidly against new diseases or against new variants of the same pathogen in case the virus mutates. When switching to develop and mass-produce a new vaccine, the genetic sequence of the antigen or candidate antigen of the viral pathogen is a product-specific prerequisite. This genetic information is then transferred into the template DNA and once the template DNA is produced, the other components of the RNA vaccine production platform technology and the QbD framework can be re-used from the previous RNA vaccine or vaccine candidate production process, thus these are agnostic to the vaccine product. Therefore, raw materials—with the exception of the template DNA—consumables, equipment, upstream and downstream unit operations, formulation components, fill-to-finish processes and quality control and quality assurance approaches can all remain unchanged when starting the production of a new vaccine or vaccine candidate. The CQAs identified in Supplementary Table 1 and the CPPs defined in Table 1 are also independent of the viral infectious disease target. The reason for this is that these CQAs and CPPs define the RNA molecule and its production process, respectively, and these CQAs and CPPs do not describe the antigen and antigen production process, as the antigen is produced in the cells of the human body based on instructions provided by the RNA molecule. Moreover, the QbD framework can be used to aid the development and manufacturing of both mRNA and saRNA vaccines, collectively referred to here as RNA vaccines.

As part of iterative model development, the QbD bioprocess model can be improved in several ways, including (1) incorporating additional CQAs and linking these to CPPs using mathematical equations, (2) adding first-principle QbD models for downstream unit operations, i.e. tangential flow filtration and chromatography purification, and (3) adapting the model for larger-scale production and purification, for example by fitting the kinetic model parameters to the RNA synthesis at larger scales or, if needed, by changing the model architecture to more accurately describe larger scale RNA synthesis. All these three model improvements are currently hindered by the lack of publicly available data since this is a new type of product and production process. An example of product CQA that can be added to the model in the future is the 5′ capping efficiency. Inclusion of the 5′ capping efficiency CQA in the current version of the model has not been prioritised because the commercially available 5′ cap analogue, CleanCap (supplied by TriLink Biotechnologies, San Diego, CA, USA) yields 5′ capping efficiencies of ≈95% which is considered high enough for the effective translation of the RNA into vaccine antigen in human cells^21–25^. In this study saRNA synthesis based on wild-type, NTPs was modelled but in future iterations, the model can also be adapted to described RNA synthesis using modified NTPs, such as N1-methylpseudouridine-5′-triphosphate^36–41^. Wild type NTPs are used for the production of Covid-19 vaccines at CureVac and at Imperial College London, whereas BioNTech/Pfizer and Moderna use modified uridine triphosphate (UTPs)^36–41^.

The mechanistic model is advantageous over statistical and data-driven models in data-scarce environments, strengthens process understanding and showcases cause–effect relationships. However, uncertainty quantification may be less robust when using mechanistic models compared to multivariate statistical modelling when sufficient experimental data is available.

A key pillar of QbD is product and process understanding. As more data becomes available, model discrimination and model-based DoE (MB-DoE)^42,43^ can be used to establish causality between CQAs and CPPs using mechanistic modelling terms. For instance, statistical DoE has established that there is an optimum in Mg concentration to maximise RNA yield. This could be the effect of a complex interplay of enzyme saturation, Mg-facilitated RNA degradation, and precipitation out of solution through magnesium PPi. All of these have different impacts on the safety and efficacy CQAs downstream. MB-DoE and stochastic Global Sensitivity Analysis could then be used to pinpoint the most probable reason. Thereafter, including additional measurements such as NTP concentration and solution turbidity measurements throughout the course of the reaction could be used to infer the most likely physical cause. With the current model, one cannot dismiss model parameters without jeopardising predictive power, nor include additional terms without overfitting. As more biochemical and bioprocess knowledge become available, insignificant parameters can be fixed or constrained in parameter estimation so that more physical parameters can be investigated without overfitting the data.

Yet, due to the high cost of experiments, simultaneous mechanistic and statistical model building might not be feasible. To this end, it might be beneficial to start out with screening experiments to build a first mechanistic DS, which is able to map larger PP spaces with fewer data. As more data becomes available, it can be combined with data-driven techniques into a hybrid model to minimise plant-model mismatch. As the mechanistic model should capture most non-linearities, the data-driven technique could even be linear and relatively inexpensive.

In conclusion, a QbD qualitative methodology and quantitative mechanistic model has been developed and applied for facilitating the rapid and high-quality production of RNA vaccines against emerging infectious diseases. The new qualitative methodology identified critical PPs (CPPs) and related these to critical quality attributes (CQAs) of the RNA vaccine transcript. The mechanistic bioprocess model mapped the value of the RNA drug substance effective yield over a four-dimensional CPPs space. This way, the first DS of an RNA vaccine synthesis bioreactor was obtained facilitating the optimal control of the production process.

This QbD framework incorporates disease-agnostic prior knowledge, experimental data, production process understanding, bioprocess modelling and can serve as a “pre-qualification” for accelerating the pre-clinical and clinical development and the regulatory approval process. By combining such a QbD framework with the RNA vaccine production platform, vaccines and vaccine candidates can be produced for future outbreaks faster and at consistent high-quality. The QbD framework follows an iterative development cycle and this QbD model can be improved and implemented to enable vaccine production against pandemics substantially faster. This can be catalysed by cross-disciplinary collaboration between academia, industry and regulatory authorities.

## Methods

### CQA identification

See Supplementary Table 1.

### CPP identification and CPP–CQA interaction

To quantitatively assess the impact of PPs on CQAs, a new methodology was developed which accounts for the magnitude, direction and type of the CPP-CQA relationship. The magnitude of the impact was rated from zero (low) to three (high), and PPs rated with two or three were considered CPPs. The direction and type of CPP-CQA relationship were characterised either by a positive slope, a negative slope, or a peak behaviour, labelled with plus, minus, or plus–minus, respectively. The ratings provided in Supplementary Table 1 and Table 1 are based on experimental data, production process understanding, information from literature^27,44–48^ and expert knowledge.

### Mechanistic model

The model aims to link the grouped effective RNA yield CQA to 4 CPPs. To relate RNA concentration to total initial NTP and Mg concentration, all buffer component concentrations need to be tracked. It is assumed that the main free species present in solution affecting transcription and degradation kinetics are: Mg^2+^, NTP^4−^, H^+^, HEPES^−^ (buffer) and PPi^4−^. These five free solution components can form the following ten complexes: HNTP^3−^, MgNTP^2−^, Mg_2_NTP, MgHNTP^−^, MgPPi^2+^, Mg_2_PPi, HPPi^3−^, H2PPi^2−^, MgHPPi^−^ and HEPES. This system naturally gives rise to differential-algebraic equations (DAE) (Eqs. (1) to (7)). The differential equations describe the variation of the total solution component concentrations using: (a) transcription term (Eq. (8)) modified from^20^, (b) a degradation term^48^ (Eq. (9)) and (c) a precipitation term (Eq. (10)). Algebraic equations then give the solution and complex concentrations through mass balance and equilibrium considerations^20^ (Equations (M1)–(M5) and (E1)–(E10) under Model equations in Supplementary Information). It is assumed that: (1) temperature is constant, (2) the DNA template has the correct sequence, (3) 5′ RNA cap analogue does not change the mechanisms of the synthesis hence its concentration is neglected, (4) the four NTP^4−^ concentrations are equimolar and (5) DTT and spermidine concentrations remain at optimal values throughout the reaction.

1 $$\frac{{d\left[ {\rm{RNA}} \right]_{\rm{tot}}}}{{dt}} = V_{tr} - V_{\rm{deg}}$$

2 $$\frac{{d\left[ {\rm{PPi}} \right]_{\rm{tot}}}}{{dt}} = \left( {N_{\rm{all}} - 1} \right) \ast V_{tr} - V_{\rm{precip}}$$

3 $$\frac{{d\left[ {\rm{NTP}} \right]_{\rm{tot}}}}{{dt}} = - N_{\rm{all}} \ast V_{tr}$$

4 $$\frac{{d\left[ H \right]_{\rm{tot}}}}{{dt}} = \left( {N_{\rm{all}} - 1} \right) \ast V_{tr}$$

5 $$\frac{{d[\rm{T7RNAP}]_{tot}}}{{dt}} = - k_d \ast \left[ {\rm{T7RNAP}} \right]_{\rm{tot}}$$

6 $$\frac{{d[\rm{Mg}]_{tot}}}{{dt}} = - 2 \ast V_{\rm{precip}}$$

7 $$\frac{{d[\rm{HEPES}]_{\rm{tot}}}}{{dt}} = 0$$

8 $$V_{tr} = k_{\rm{app}} \ast \left[ {\rm{T7RNAP}} \right]_{\rm{tot}}\frac{{\left[ {\rm{Mg}} \right]\,\left[ {\rm{MgNTP}} \right]}}{{1 + K_1\left[ {\rm{Mg}} \right] + K_2\left[ {\rm{MgNTP}} \right]}}$$

9 $$V_{\rm{deg}} = (k_{\rm{Ac}}\left[ H \right]^{n_{\rm{ac}}}\, +\, k_{\rm{ba}}\left[ {\rm{OH}} \right]^{n_{\rm{ba}}} \,+\, k_{\rm{Mg}}\left[ {\rm{Mg}} \right]^{n_{\rm{Mg}}})\left[ {\rm{RNA}} \right]^{n_{\rm{RNA}}}$$

10 $$V_{\rm{precip}} = {\mathrm{max}}(0,\,k_{\rm{precip}}\left( {\left[ {\mathrm{Mg}}_2{\mathrm{PPi}} \right] - \left[ {\mathrm{Mg}}_2{\mathrm{PPi}} \right]_{\rm{eq}}} \right))$$

### Model implementation

The system of DAEs was solved explicitly by breaking up the problem into two: (1) The differential equations expressing the total solution components were solved as initial value problems using an in-house fourth-order Runge–Kutta solver. The initial conditions of the ODE system are set to 0 M for RNA, 0.04 M for HEPES, the equivalent of 7.5 pH for total protonated components and 1 × 10^−18^ M PPi (nonzero for numerical stability), for further details see the SI document. The initial concentrations of total Mg, NTP and T7RNAP depend on the model input. (2) The solution concentrations that appeared in the kinetic terms were solved for at each time step using scipy.optimise.fsolve().

### Parameter estimation

The model was fitted and validated with a set of 51 experimental data samples with three replicates each obtained from saRNA synthesis experiments using wild-type, non-modified UTPs^28^. Biological knowledge was used to set *N*_all_, the length of the RNA chain, to 10,000 bases. Similarly, [Mg_2_PPi]_eq_ was found to be 1.4 × 10^−5^ mol/L and the values of dissociation equilibrium constants were taken to be ${{10}}^{{{ - 6}}{{.95}}},\,{{10}}^{{{ - 4}}{{.42}}},\,{{10}}^{ - {{1}}{{.69}}},\,{{10}}^{ - {{1}}{{.49}}},\,{{10}}^{ - {{5}}{{.42}}},\,{{10}}^{ - {{2}}{{.33}}},\,{{10}}^{ - {{8}}{{.94}}},\,{{10}}^{ - {{6}}{{.13}}},\,{{10}}^{ - {{3}}{{.05}}},\,{{10}}^{ - {{7}}{{.5}}}$ mol/L for *K*_eq,0_ to *K*_eq,9_ respectively. To reduce overfitting, *n*_ac_, *n*_ba_, *n*_Mg_ and *n*_RNA_ were set to 1. The phenomena of enzyme degradation and Mg_2_PPi precipitation were ignored for now, and hence *k*_d_ and *k*_precip_ set to 0, as they did not improve model performance. The residual six parameters *k*_app_, *K*_1_, *K*_2_, *k*_ac_, *k*_ba_ and *k*_Mg_ were then estimated using the scipy.optimise.*curve*_*fit()* local solver function in Python 3 through least-squares error minimisation, with initial guesses *k*_app_ 1.3 × 10^−3^ $\frac{\rm{L}^2}{\rm{mol}\,\rm{U}\,\rm{h}}$, *K*_1_ 20 $\frac{\rm{L}}{\rm{mol}}$, *K*_2_ 100 $\frac{\rm{L}}{\rm{mol}}$, *k*_ac_ 1 × 10^6^ $\frac{\rm{L}}{\rm{mol}\,\rm{h}}$, *k*_ba_ 1 × 10^6^ $\frac{\rm{L}}{\rm{mol}\,\rm{h}}$ and *k*_Mg_ 2 $\frac{\rm{L}}{\rm{mol}\,\rm{h}}$.

### Sensitivity analysis

The described model was implemented in gPROMS (Process Systems Enterprise, London, UK), in which the Global Systems Analysis entity was used to perform variance-based sensitivity analysis. Therein, 80,000 simulations were run at 0.075 M Mg, 0.04 M NTP and 1 × 10^−8^ M T7RNAP where the kinetic parameters were quasi-randomly generated, using Sobol sequences, in a uniformly distributed range at ±10% around the optimal kinetic parameter values which were generated using parameter estimation.

### Statistical models

The 51 averaged data samples from the parameter estimation were uploaded to the MODDE^®^ statistical Design of Experiments software. Time, initial Mg, initial NTP and initial T7RNAP concentrations were included as factors and scaled to unit variance. Then, two separate models were fitted using MLR, one using the four factors as linear predictors and one that also included square terms in the Mg and NTP concentrations as well as an interaction term consisting of the product of Mg and NTP concentrations.

### Probabilistic DS

For determining the probabilistic 2D DS, each Mg/NTP point was Monte-Carlo simulated 50 times using a random normal uncertainty, with a standard deviation of 20% around the optimal kinetic rate constant model parameters.

### Cost analysis

GMP grade T7RNAP and wild-type, unmodified NTP costs were obtained from Roche Diagnostics International Ltd. as 1.35 × 10^8^ $/mol and 2.5 × 10^5^ $/mol, respectively. These cost values are representative of these products and over time it is expected that these raw material purchase prices will decrease due to technology maturation and economies of scale, as the RNA vaccine platform technology will be used to produce other vaccine product leading to an increased demand for these raw materials.

### Reporting summary

Further information on research design is available in the Nature Research Reporting Summary linked to this article.

## Supplementary information

Supplementary Information

Reporting Summary

## Data Availability

Experimental data are available from ref. ^28^. These data were obtained from saRNA synthesis experiments using wild-type, non-modified UTPs^28^. The model was calibrated using this data.

## Code availability

The documented code is available online at https://github.com/dv516/RNA-transcription-modelling-and-DS.
