# Supplementary material for: Quality by design modelling to support rapid RNA vaccine production against emerging infectious diseases
Source: NPJ Vaccines. 2021 Apr 29;6:65. doi: 10.1038/s41541-021-00322-7 (PMC8085199; doi:10.1038/s41541-021-00322-7)
Supplement: Supplementary file 1 — Supplementary Information [file 41541_2021_322_MOESM1_ESM.pdf]

# Quality by Design modelling to support rapid RNA vaccine production against emerging infectious diseases

## Supplementary Information

Damien van de Berg[1], Zoltán Kis[1], Carl Fredrik Behmer[1], Karnyart Samnuan[2], Anna K. Blakney[2], Cleo Kontoravdi[1], Robin Shattock[2], Nilay Shah[1]

[1]Centre for Process Systems Engineering, Department of Chemical Engineering, Faculty of Engineering, Imperial College London, SW7 2AZ London, UK.
[2]Department of Infectious Disease, Faculty of Medicine, Imperial College London, W2 1PG London, UK.

**Supplementary Table 1.** Assessment of the criticality of quality attributes based on the methodology presented in the A-Vax study [1]. The criticality of quality attributes is assessed based on the severity score which is obtained by the multiplication of the impact score by the uncertainty score both for safety and efficacy. Attributes with severity scores of below 10 are considered non-critical, attributes with severity scores of 25, 50 and 75 are considered critical and attributes with severity scores of 16, 24, 32, 40, 100 and 125 are considered potentially critical.

| QAs | Unit | Acceptance criteria | Impact Score - Safety$^{\alpha}$ | Impact Score - Efficacy$^{\alpha}$ | Uncertainty score - Safety$^{\beta}$ | Uncertainty score - Efficacy$^{\beta}$ | Severity Score – Safety$^{\gamma}$ | Severity Score – Efficacy$^{\delta}$ | Max Severity | Classification$^{\varepsilon}$ |
|---|---|---|---|---|---|---|---|---|---|---|
| Amount of precipitate | g/L | <0.001 | 2 | 8 | 4 | 4 | 8 | 32 | 32 | pCQA |
| RNA sequence integrity | kb (length) | >9 | 8 | 25 | 4 | 3 | 32 | 75 | 75 | CQA |
| RNA sequence identity | %match | >99% | 8 | 25 | 4 | 3 | 32 | 75 | 75 | CQA |
| RNA yield | g/L | >1.5 | 2 | 8 | 3 | 3 | 6 | 24 | 24 | CQA |
| 5' capping efficiency | % | >85% | 2 | 25 | 3 | 2 | 6 | 50 | 50 | CQA |
| Residual host cell proteins (E. Coli) | g/L | <500 ng/mg RNA | 2 | 2 | 3 | 3 | 6 | 6 | 6 | QA |
| Residual host cell DNA (E. Coli) | g/L | <100 ng/mg RNA | 8 | 2 | 3 | 3 | 24 | 6 | 24 | QA |
| Residual template DNA | g/L | <50 ng/mg RNA | 8 | 2 | 3 | 3 | 24 | 6 | 24 | QA |
| Bacterial endotoxins | g/L | None | 25 | 2 | 2 | 4 | 50 | 8 | 50 | CQA$^{\omega}$ |
| Bioburden | g/L | None | 25 | 8 | 2 | 4 | 50 | 32 | 50 | CQA$^{\omega}$ |
| Post-filtration pH | / | ±0.1 | 2 | 25 | 3 | 3 | 6 | 75 | 75 | CQA$^{\omega}$ |
| Salt concentration | mM | ±0.1 | 2 | 8 | 3 | 3 | 6 | 24 | 24 | QA |

$^{\alpha}$ Impact score, assesses the impact of the quality attribute on product safety and/or efficacy for the patients. Scored with 2 for low, 8 for moderate and with 25 for high.

$^{\beta}$ Uncertainty score, assesses the level of uncertainty of the impact of the attribute on safety and/or efficacy for the patients. Scored with 1 for minimal, 2 for low, 3 for moderate, 4 for high, and 5 for very high.

$^{\gamma}$ Severity Score for safety is obtained by the multiplication of the Impact Score for safety by the Uncertainty Score for safety.

$^{\delta}$ Severity Score for efficacy is obtained by the multiplication of the Impact Score for efficacy by the Uncertainty Score for efficacy.

[ε] QA – non-critical quality attribute (marked in green), due to low impact scores of 2 or moderate impact score of 8 and resulting severity score values of 2, 4, 6, or 8; CQA (marked in red) – critical quality attribute, due to high impact score of 25 and uncertainty score of below 3, resulting in severity score values of 25, 50, 75; pCQA (marked in yellow)– potential critical quality attribute, the remaining severity score values, due to high impact scores of 8 or 25 and uncertainty score of below 2-5, resulting in severity score values of 16, 24, 32, 40, 100, 125.
[ω] These CQAs are well-controlled in a GMP bioproduction process, hence these QCAs were not included in the model.

The concentration of the 5’ cap analogue remains unchanged when RNA vaccines of different length are used due to the very high molar excess of the 5’ cap analogue used relative to the final molar RNA concentration; the calculations are available in **Supplementary Table 2**.

For example, when producing a 2 kilobases long mRNA the excess of the CleanCap AG reagent is over 99.87% and when producing a 10 kilobases long saRNA the excess of the CleanCap AU reagent is over 99.97%. Additionally, the 5’ cap analogue supplier recommends the identical starting CleanCap AG or CleanCap AU concentrations in the reaction mix when producing mRNA or saRNA transcripts, respectively, at similar RNA transcript yields and capping efficiencies, but different transcript lengths [43,44]. Therefore, due to its high molar excess, the initial and final 5’ cap analogue concentration in the reaction mix remains largely unchanged when producing 5 g per L of ≈95% capped transcripts of different lengths [43,44]. Moreover, the purchase price of the 5’ cap analogue remains unchanged when the length of RNA transcript changes, because the relative purchase price of the CleanCap AG compared to CleanCap AU depends largely on the purchase amounts due to the economies of scales which influence the production of these 5’ cap analogues.

**Supplementary Table 2**. Calculation of CleanCap AG and CleanCap AU molar concentration excesses with respect to molar concentration of RNA transcripts of different length.

| Parameter name | Parameter Symbol | Calculation formula or Reference | Parameter Value | Parameter Unit |
|---|---|---|---|---|
| Average NTP molecular mass | $MM_{NTP}$ | Average of molecular mass of the 4 NTPs (ATP, CTP, GTP and UTP) constituting RNA molecules.* | 499.414 | Da or g × $Mol^{-1}$ |
| Number of NTPs in a 10 kb RNA | $N_{NTP\ 10kb\ RNA}$ | Count of the number of bases in a 10 kilobases RNA polymer | 10,000 | Number of nucleobases |
| Average molecular mass of a 10 kb RNA | $MM_{10kb\ RNA}$ | $MM_{10kb\ RNA} = N_{NTP\ 10kb\ RNA} \times MM_{NTP}$ | 4,994,142.5 | Da or g × $Mol^{-1}$ |
| Number of NTPs in a 2 kb RNA | $N_{NTP\ 2kb\ RNA}$ | Count of the number of bases in a 2 kilobases RNA polymer | 2,000 | Number of nucleobases |
| Average molecular mass of a 2 kb RNA | $MM_{2kb\ RNA}$ | $MM_{2kb\ RNA} = N_{NTP\ 2kb\ RNA} \times MM_{NTP}$ | 998,828.5 | Da or g × $Mol^{-1}$ |
| CleanCap AU or CleanCap AG concentration in the reaction mix | $CM_{CleanCap}$ | [2–4] | 0.004 | M or moles × $L^{-1}$ |
| Avogadro's constant | $N_A$ | [5] | $6.02214076 \times 10^{23}$ | Molecules × $mol^{-1}$ |
| Number of CleanCap AU or CleanCap AG molecules per L | $N_{CleanCap}$ | $N_{CleanCap} = CM_{CleanCap} \times N_A$ | $2.40885630 \times 10^{21}$ | Molecules × $L^{-1}$ |
| Yield of the 10 kb RNA transcript in the reaction mix | $Y_{10kb\ RNA}$ | [2–4] | 5 | g × $L^{-1}$ |
| Yield of the 2 kb RNA transcript in the reaction mix | $Y_{2kb\ RNA}$ | [2–4] | 5 | g × $L^{-1}$ |
| Molar concentration of the 10 kb RNA in the reaction mix | $CM_{10kb\ RNA}$ | $CM_{10kb\ RNA} = Y_{10kb\ RNA} \times MM_{10kb\ RNA}^{-1}$ | $1.00117287 \times 10^{-6}$ | M or moles × $L^{-1}$ |
| Molar concentration of the 2 kb RNA in the reaction mix | $CM_{2kb\ RNA}$ | $CM_{2kb\ RNA} = Y_{2kb\ RNA} \times MM_{2kb\ RNA}^{-1}$ | $5.00586437 \times 10^{-6}$ | M or moles × $L^{-1}$ |
| Number of 10 kb RNA molecules per L | $N_{10kb\ RNA}$ | $N_{10kb\ RNA} = CM_{10kb\ RNA} \times N_A$ | $6.02920397 \times 10^{17}$ | Molecules × $L^{-1}$ |
| Number of 2 kb RNA molecules per L | $N_{2kb\ RNA}$ | $N_{2kb\ RNA} = CM_{2kb\ RNA} \times N_A$ | $3.01460199 \times 10^{18}$ | Molecules × $L^{-1}$ |
| **CleanCap AU excess** | $E_{CleanCap\ AU}$ | $E_{CleanCap\ AU} = (N_{CleanCap} - N_{10kb\ RNA}) \times 100 \times N_{CleanCap}^{-1}$ | **99.9750** | **%** |
| **CleanCap AG excess** | $E_{CleanCap\ AG}$ | $E_{CleanCap\ AG} = (N_{CleanCap} – N_{2kb\ RNA}) \times 100 \times N_{CleanCap}^{-1}$ | **99.8749** | **%** |

* Average molecular mass of NTPs calculated by taking the average of the 4 NTPs found in RNA sequences, assuming equimolar ratios of the 4 NTPs. The molecular masses of ATP, CTP, GTP and UTP are 507.18, 483.156, 523.18 and 484.141 g × $Mol^{-1}$, respectively.

**Supplementary Table 3**. Factor Sensitivity Table. The first order and total effect Sobol indices of the RNA yield response with respect to the six estimated model parameters is given after 6 hours of reaction time and as an average over the first 8 hours of the reaction. These results were obtained using global variance-based sensitivity analysis sampling 80,000 realisations of an experimental run at initial conditions of 0.075 M Mg, 0.04 M NTP and $1\times10^{-8}$ M T7RNAP from uniform distributions of ±10% around the optimal kinetic model parameter values from the parameter estimation.

| Kinetic parameter* | **RNA yield after 6 hours of *in vitro* transcription reaction time** | | **Average RNA yield over the first 8 hours of *in vitro* transcription reaction** | |
|---|---|---|---|---|
| | **First-order effect** | **Total-order effect** | **First-order effect** | **Total-order effect** |
| **k_app** | 0.613937828 | 0.615556387 | 0.623147208 | 0.624923921 |
| **K_1** | 0.300884499 | 0.302357491 | 0.303372512 | 0.305001923 |
| **K_2** | 0.056224231 | 0.05663848 | 0.058120643 | 0.05862554 |
| **k_ac** | 0.026960587 | 0.027121538 | 0.013332301 | 0.01340837 |
| **k_ba** | 4.21E-05 | 3.91E-29 | 9.74E-06 | 5.20E-29 |
| **k_Mg** | 3.65E-04 | 3.34E-04 | 1.50E-04 | 1.46E-04 |

*The kinetic model parameters have been quasi-randomly varied using Sobol sequences within a uniformly distributed range of ±10% around the optimal parameter values obtained from the parameter estimation. The impact of each quasi-randomly generated parameter value on the RNA yield has been determined using variance-based global sensitivity analysis and is illustrated by the First-order effect and the Total-order effect [6–10].

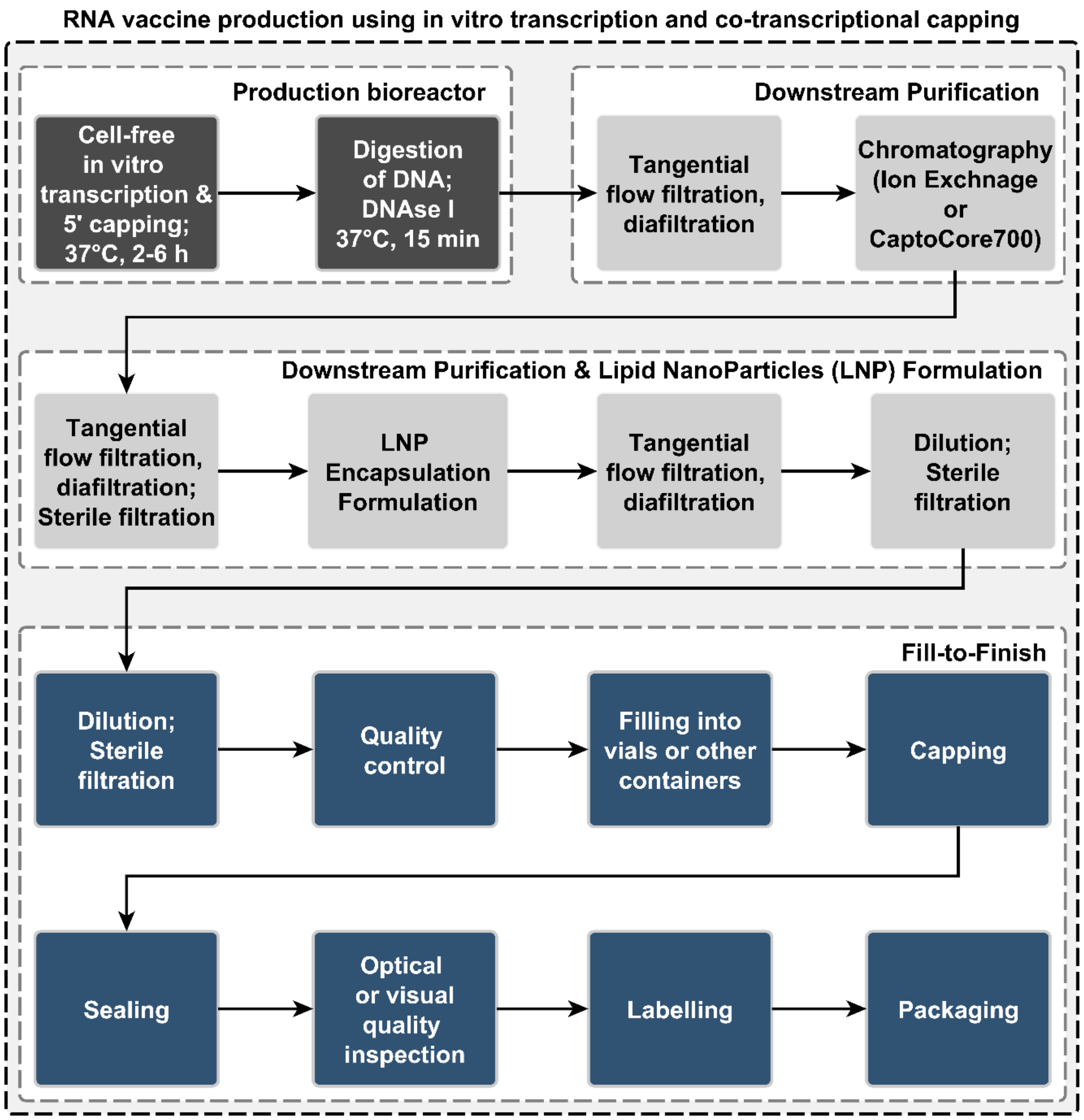
RNA vaccine production using in vitro transcription and co-transcriptional capping
Production bioreactor
Cell-free in vitro transcription & 5' capping; 37°C, 2-6 h
Digestion of DNA; DNAse I 37°C, 15 min
Downstream Purification
Tangential flow filtration, diafiltration
Chromatography (Ion Exchnage or CaptoCore700)
Downstream Purification & Lipid NanoParticles (LNP) Formulation
Tangential flow filtration, diafiltration; Sterile filtration
LNP Encapsulation Formulation
Tangential flow filtration, diafiltration
Dilution; Sterile filtration
Fill-to-Finish
Dilution; Sterile filtration
Quality control
Filling into vials or other containers
Capping
Sealing
Optical or visual quality inspection
Labelling
Packaging

**Supplementary Figure 1**. Process flow diagram for RNA drug substance production (aka. active ingredient production, bulk production or primary manufacturing) and drug product manufacturing (aka. fill-to-finish or secondary manufacturing). The RNA drug substance is synthesized in the production bioreactor using the *in vitro* transcription reaction with the T7 RNA polymerase enzyme and is 5' capped co-transcriptionally. After RNA synthesis, the template DNA is digested using the DNAse I enzyme. Next, the downstream purification starts with tangential flow filtration (TFF) where the RNA molecule is retained by the filter and the other, smaller components of the reaction mix flow through the TFF filter. Next, the protein enzymes can be further removed using a chromatography unit operation, such as ion exchange chromatography, CaptoCore 700 chromatography, or hydroxyapatite chromatography. Next, the buffer is replaced for the formulation buffer in a second TFF step and then the solution is sterile filtered. This solution then enters the lipid nanoparticle (LNP) encapsulation unit operation which is the bottleneck of RNA drug substance production. After formulation, the LNP encapsulated RNA solution enters a third TFF for diafiltration then an optional dilution step is carried out followed by a sterile filtration operation. The sterile LNP-encapsulated RNA solution is then transferred to the fill-to-finish section. There, the solution can be further diluted and sterile filtered. Next, the formulated RNA solution undergoes quality control and is filled into vials or other containers. The vials are then capped, sealed, inspected, labelled and packaged into secondary and tertiary packaging [11–18].

The scatter plots obtained from the variance-based global sensitivity analysis results are shown below in **Supplementary Figure 2**. The 80,000 dots in each scatter plot were obtained by running 80,000 simulations at 0.075 M Mg, 0.04 M NTP and $1\times10^{-8}$ M T7RNAP initial concentrations where the kinetic parameters were quasi-randomly varied in a uniformly distributed range at ±10% around the optimal kinetic parameter values which were obtained using parameter estimation. The quasi-random variation of the model parameters was obtained using Sobol sequences. If the dots of the scatter plots are clustered around a narrow range of values on the y-axis, for example resembling a line, then the input parameter on the x-axis describes the variation of the output parameter plotted on the y-axis. On the other hand, if the dots are scattered across a wide range of y-values, the variability is explained by other input parameters.

A

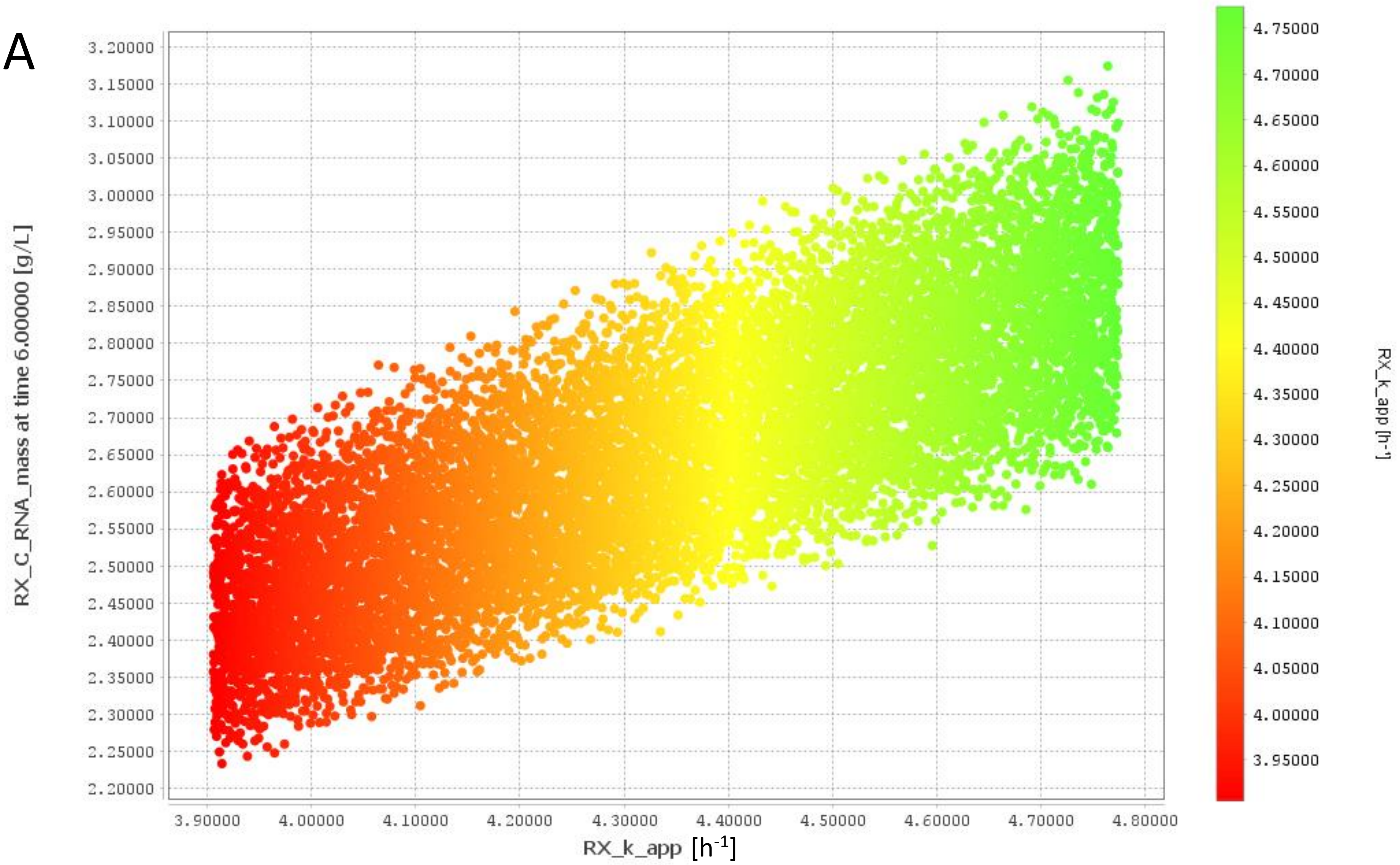

RX_C_RNA_mass at time 6.00000 [g/L]
3.20000
3.15000
3.10000
3.05000
3.00000
2.95000
2.90000
2.85000
2.80000
2.75000
2.70000
2.65000
2.60000
2.55000
2.50000
2.45000
2.40000
2.35000
2.30000
2.25000
2.20000
3.90000
4.00000
4.10000
4.20000
4.30000
4.40000
4.50000
4.60000
4.70000
4.80000
RX_k_app [h-1]
4.75000
4.70000
4.65000
4.60000
4.55000
4.50000
4.45000
4.40000
4.35000
4.30000
4.25000
4.20000
4.15000
4.10000
4.05000
4.00000
3.95000
RX_k_app [h-1]

B

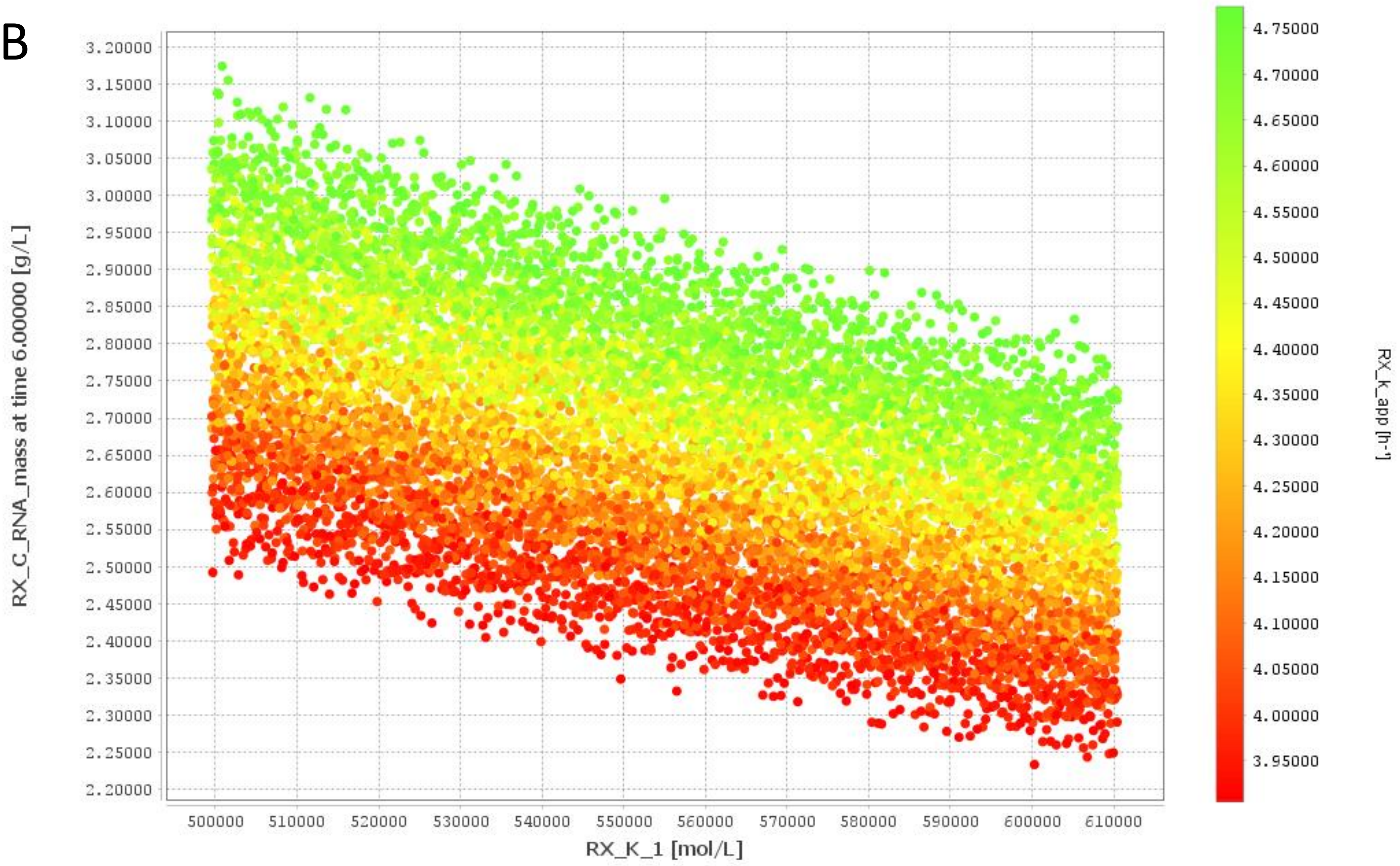

3.20000
3.15000
3.10000
3.05000
3.00000
2.95000
2.90000
2.85000
2.80000
2.75000
2.70000
2.65000
2.60000
2.55000
2.50000
2.45000
2.40000
2.35000
2.30000
2.25000
2.20000
RX_C_RNA_mass at time 6.00000 [g/L]
500000
510000
520000
530000
540000
550000
560000
570000
580000
590000
600000
610000
RX_K_1 [mol/L]
4.75000
4.70000
4.65000
4.60000
4.55000
4.50000
4.45000
4.40000
4.35000
4.30000
4.25000
4.20000
4.15000
4.10000
4.05000
4.00000
3.95000
RX_k_app [h-1]

C

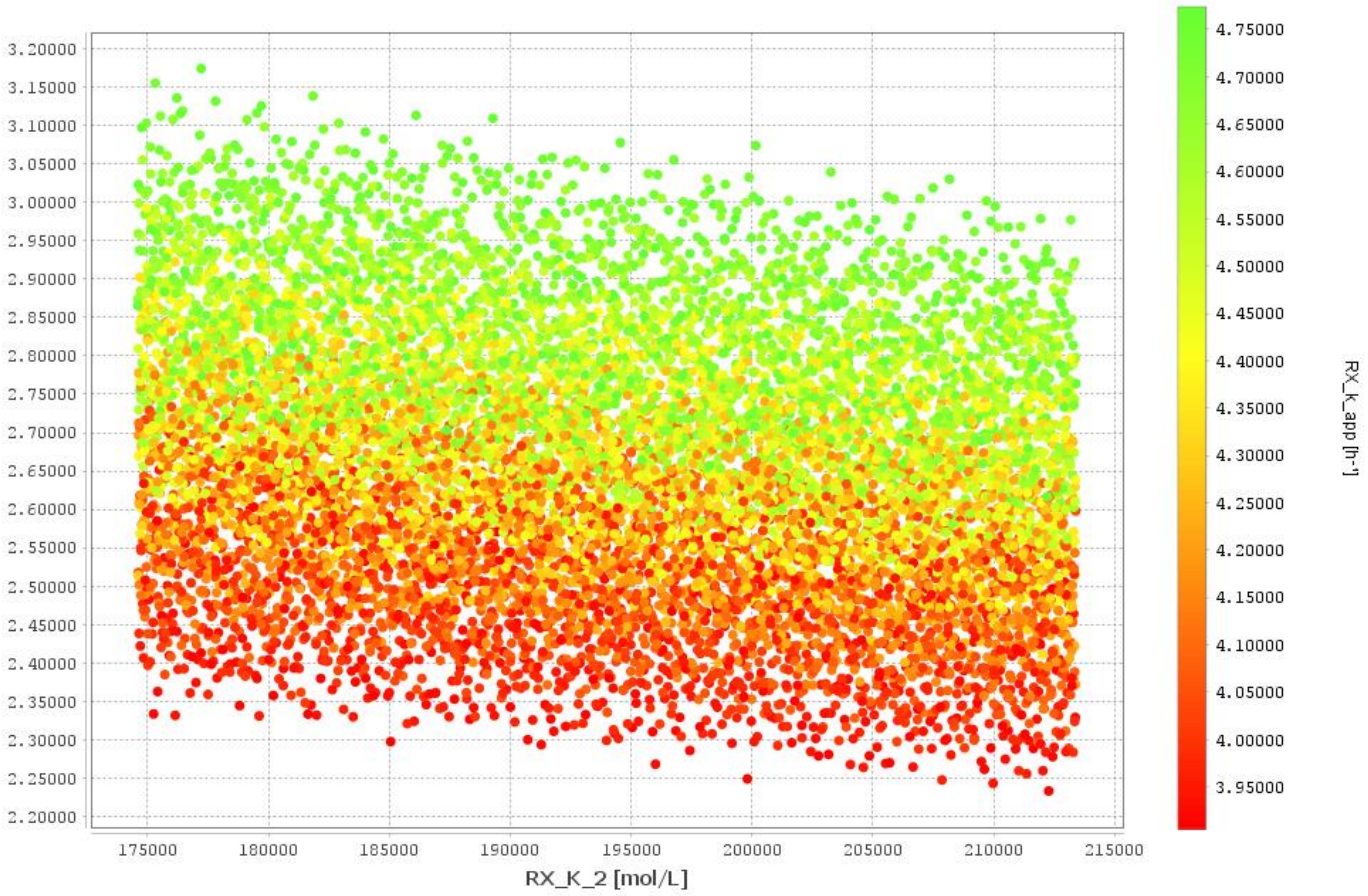

RX_C_RNA_mass at time 6.00000 [g/L]
3.20000
3.15000
3.10000
3.05000
3.00000
2.95000
2.90000
2.85000
2.80000
2.75000
2.70000
2.65000
2.60000
2.55000
2.50000
2.45000
2.40000
2.35000
2.30000
2.25000
2.20000
175000
180000
185000
190000
195000
200000
205000
210000
215000
RX_K_2 [mol/L]
4.75000
4.70000
4.65000
4.60000
4.55000
4.50000
4.45000
4.40000
4.35000
4.30000
4.25000
4.20000
4.15000
4.10000
4.05000
4.00000
3.95000
RX_k_app [h-1]

D

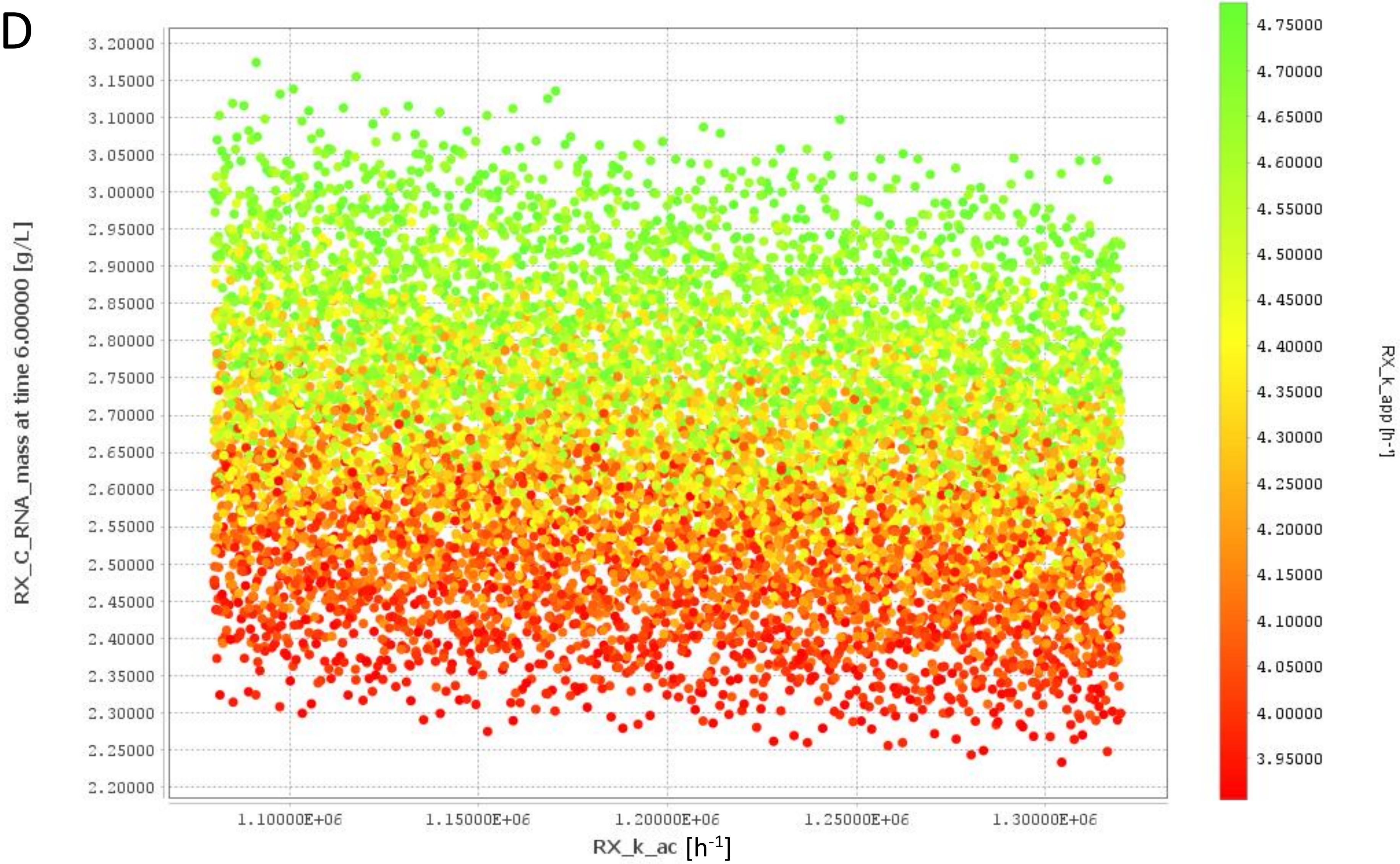

3.20000
3.15000
3.10000
3.05000
3.00000
2.95000
2.90000
2.85000
2.80000
2.75000
2.70000
2.65000
2.60000
2.55000
2.50000
2.45000
2.40000
2.35000
2.30000
2.25000
2.20000
RX_C_RNA_mass at time 6.00000 [g/L]
1.10000E+06
1.15000E+06
1.20000E+06
1.25000E+06
1.30000E+06
RX_k_ac [h-1]
4.75000
4.70000
4.65000
4.60000
4.55000
4.50000
4.45000
4.40000
4.35000
4.30000
4.25000
4.20000
4.15000
4.10000
4.05000
4.00000
3.95000
RX_k_app [h-1]

E

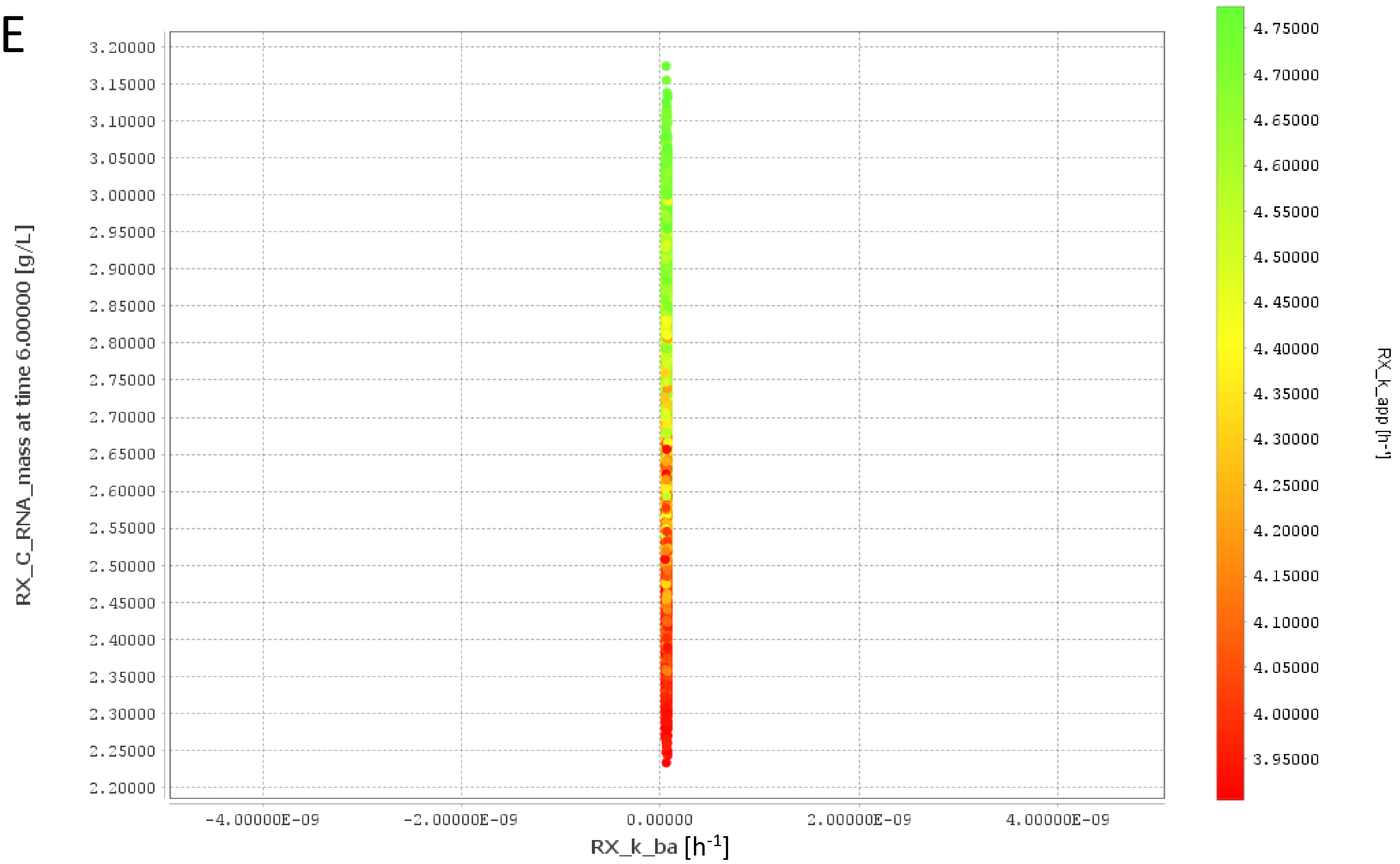
RX_C_RNA_mass at time 6.00000 [g/L]
3.20000
3.15000
3.10000
3.05000
3.00000
2.95000
2.90000
2.85000
2.80000
2.75000
2.70000
2.65000
2.60000
2.55000
2.50000
2.45000
2.40000
2.35000
2.30000
2.25000
2.20000
-4.00000E-09
-2.00000E-09
0.00000
2.00000E-09
4.00000E-09
RX_k_ba [h-1]
4.75000
4.70000
4.65000
4.60000
4.55000
4.50000
4.45000
4.40000
4.35000
4.30000
4.25000
4.20000
4.15000
4.10000
4.05000
4.00000
3.95000
RX_k_app [h-1]

F

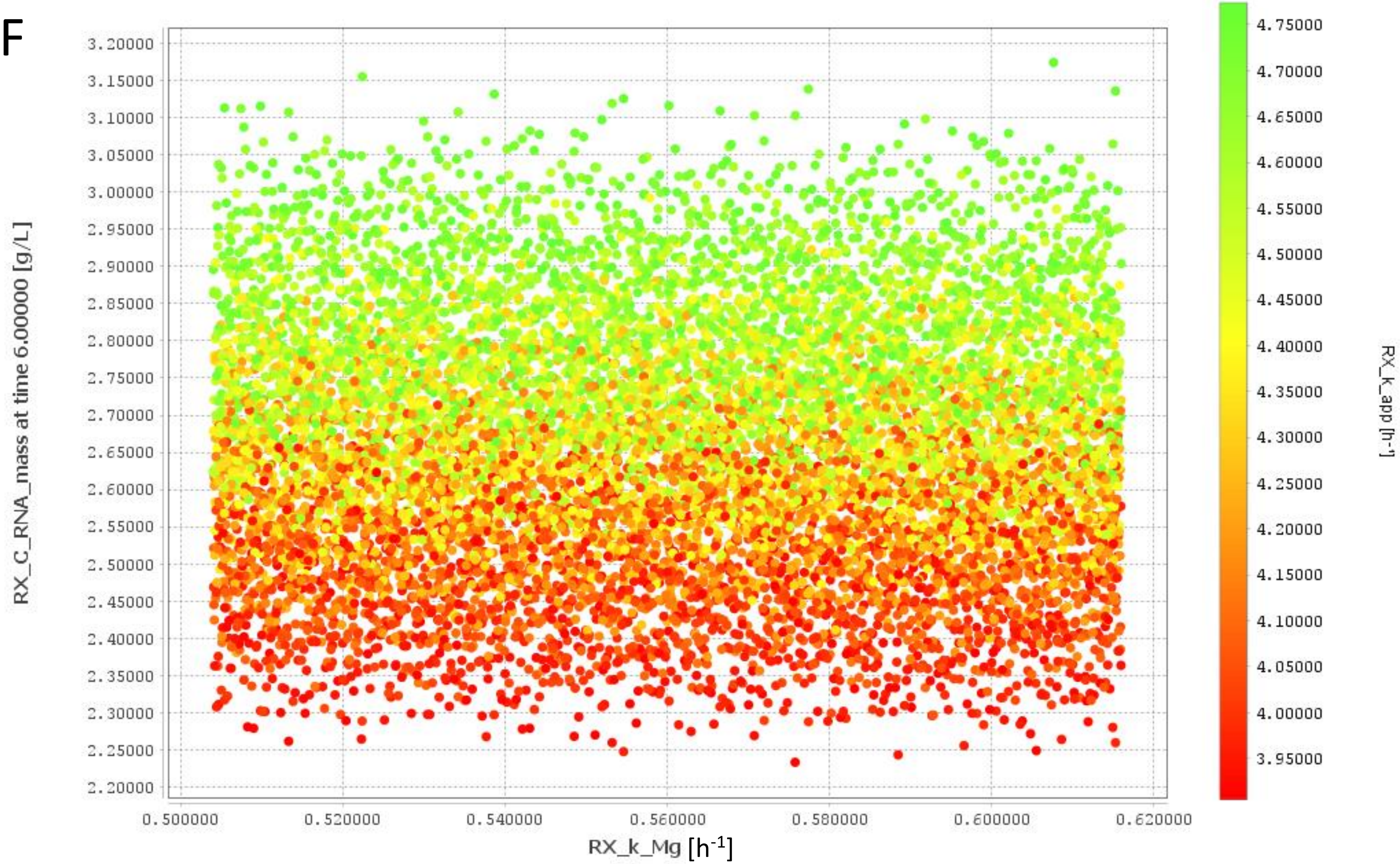
RX_C_RNA_mass at time 6.00000 [g/L]
RX_k_Mg [h-1]
RX_k_app [h-1]
3.20000
2.20000
0.500000
0.620000
4.75000
3.95000

**Supplementary Figure 2**. Sensitivity Scatter plots. The figures show the RNA yield after 6 hours from global sensitivity analysis sampling 80,000 realisations of an experimental run at initial concentrations of 0.075 M Mg, 0.04 M NTP and $1\times10^{-8}$ M T7RNAP from uniform distributions of ±10% around the optimal parameter values from the parameter estimation. **A.** Scatter plots of RNA yield after 6 hours of transcription with respect to the value of parameter $k_{app}$ for different realisation of $K_1$ , $K_2$ , $k_{ac}$ , $k_{ba}$, $k_{Mg}$. **B.** Scatter plots of RNA yield after 6 hours of transcription with respect to the value of parameter $K_1$ for different realisation of $k_{app}$ , $K_2$ , $k_{ac}$ , $k_{ba}$, $k_{Mg}$. **C.** Scatter plots of RNA yield after 6 hours of transcription with respect to the value of parameter $K_2$ for different realisation of $k_{app}$ ,$K_1$ , $k_{ac}$ , $k_{ba}$, $k_{Mg}$. **D.** RNA yield scatter plots after 6 hours of transcription with respect to the value of parameter $k_{ac}$ for different realisation of $k_{app}$ ,$K_1$ , $K_2$ , $k_{ba}$, $k_{Mg}$. **E.** Scatter plots of RNA yield after 6 hours of transcription with respect to the value of parameter $k_{ba}$ for different realisation of $k_{app}$ , $K_1$ , $K_2$ , $k_{ac}$, $k_{Mg}$. **F.** RNA yield scatter plots after 6 hours of transcription with respect to the value of parameter $k_{Mg}$ for different realisation of $k_{app}$, $K_{1,}$ $K_2$ , $k_{ac}$ , $k_{ba}$. The plots are color-coded according to $k_{app}$, the only model parameter driving the reaction forward.

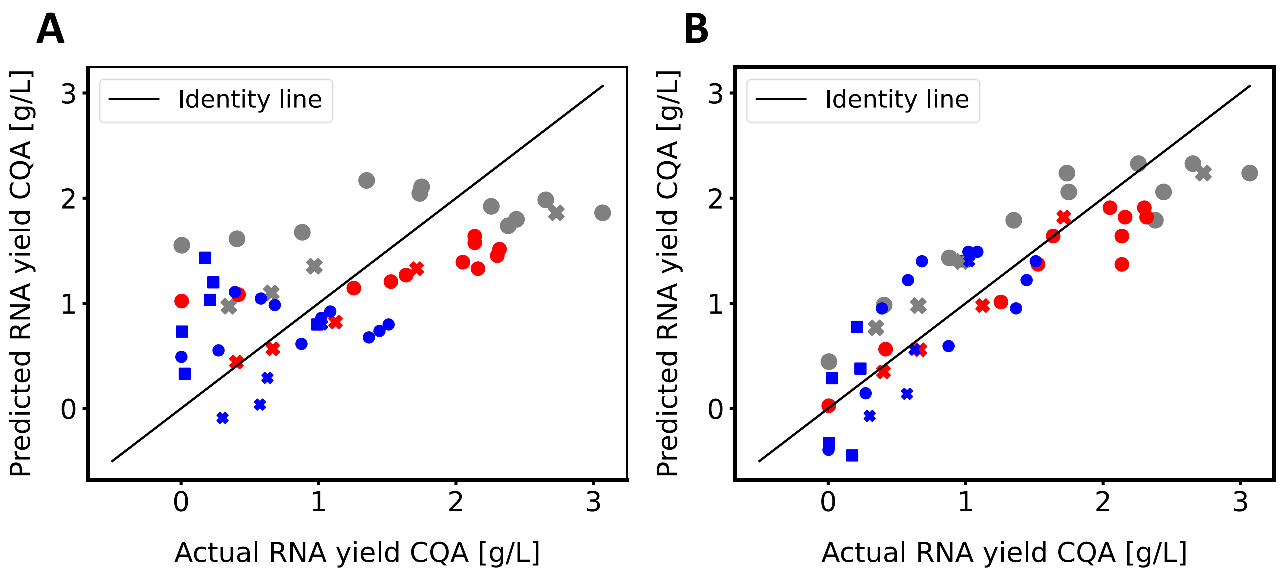


**Supplementary Figure 3**. Prediction error plots of MLR statistical models. The x-axis represents the true experimental RNA yield, and the y-axis marks the corresponding prediction from the model. Each point (circle, square or x) is a prediction generated using the model. The black line represents the identity line, where modelling results perfectly match the experimental outcome. **A**. Multiple linear regression (MLR) fit using only the four linear predictors time, initial Mg concentration, initial NTP concentration and initial T7RNAP concentration achieving an $R^2$ value of 0.398 and a mean absolute error of 0.570 g/L with 4 coefficients and a constant. **B**. Data fitting based on linear terms of the model from plot A as well as squared and interaction terms in Mg and NTP achieving an $R^2$ value of 0.766 and a mean absolute error of 0.167 g/L.

**Supplementary Table 4**. Worksheet statistics. This table gives the statistics of the RNA yield observations of the experimental dataset. The table was produced alongside **Supplementary Table 5** and **Supplementary Table 6** as part of the descriptive statistics describing the respective MLR fits.

| | **RNA yield** |
|---|---|
| Worksheet statistics | |
| Worksheet runs | 51 |
| N | 51 |
| Min | 0.00226667 |
| Max | 3.06667 |
| Mean | 1.15453 |
| Q(25%) | 0.4045 |
| Q(75%) | 1.74334 |
| Median | 1.02 |
| Std. dev. | 0.853383 |
| Min/Max | 0.000739131 |
| Std. dev./Mean | 0.739161 |
| Skewness | 0.421131 |
| Skewness test | 1.2629 |
| Kurtosis | -0.939453 |

**Supplementary Table 5**. Linear MLR model statistics. This table shows the descriptive statistics of the MLR fit predicting RNA yield using linear terms of time, initial Mg concentration, initial NTP concentration and initial T7RNAP concentration. Thus, using 4 coefficients and a constant in MLR yielded a fit with an $R^2$ value of 0.398 and a mean absolute error of 0.570 g/L.

| **Model statistics** | |
|---|---|
| Model type | Evaluation of MLR model |
| Scaling type | All factors are orthogonally scaled |
| DF | 46 |
| R2 | 0.398027 |
| R2 adj | 0.345682 |
| Q2 | 0.214196 |
| Power (post-hoc) | 0.991 |
| Condition number | 1.20307 |
| Model terms | 5 |
| DF residual | 46 |
| RSD | 0.690301 |
| p model | 8.65E-05 |
| DF lack of fit | 42 |
| p lack of fit | 0.0416183 |
| DF pure error (repl. runs) | 4 |
| SD pure error | 0.284829 |
| Residual skewness | -0.401681 |
| Residual skewness test | -1.20457 |

**Supplementary Table 6**. MLR with linear, squared and interaction terms model stastistics. This table shows the descriptive statistics of the MLR fit predicting RNA yield using linear terms of time, initial Mg concentration, initial sNTP concentration and initial T7RNAP concentration as well as squared terms in initial Mg concentration, squared terms in initial NTP concentration and an interssaction term consisting of the initial Mg concentration and initial NTP concentration product. Thus, using 7 coefficients and a constant in MLR yielded a fit with an $R^2$ value of 0.766 and a mean absolute error of 0.167 g/L.

| **Model statistics** | |
|---|---|
| Model type | Evaluation of MLR model |
| Scaling type | All factors are orthogonally scaled |
| DF | 43 |
| R2 | 0.765741 |
| R2 adj | 0.727605 |
| Q2 | -0.459336 |
| Power (post-hoc) | 1 |
| Condition number | 8.13406 |
| Model terms | 8 |
| DF residual | 43 |
| RSD | 0.445393 |
| p model | 1.17E-11 |
| DF lack of fit | 39 |
| p lack of fit | 0.182395 |
| DF pure error (repl. runs) | 4 |
| SD pure error | 0.284829 |
| Residual skewness | -4.28E-06 |
| Residual skewness test | -1.28E-05 |

**Model Equations:**

$$\frac{d[RNA]_{tot}}{dt} = V_{tr} - V_{deg} \quad (Eq. 1)$$

$$\frac{d[PPi]_{tot}}{dt} = (Nall - 1) * V_{tr} - V_{precip} \quad (Eq. 2)$$

$$\frac{d[NTP]_{tot}}{dt} = -N_{all} * V_{tr} \quad (Eq. 3)$$

$$\frac{d[H]_{tot}}{dt} = (Nall - 1) * V_{tr} \quad (Eq. 4)$$

$$\frac{d[T7RNAP]_{tot}}{dt} = -k_d * [T7RNAP]_{tot} \quad (Eq. 5)$$

$$\frac{d[Mg]_{tot}}{dt} = -2 * V_{precip} \quad (Eq. 6)$$

$$\frac{d[HEPES]_{tot}}{dt} = 0 \quad (Eq. 7)$$

$$V_{tr} = k_{app} * [T7RNAP]_{tot} \frac{[Mg]\,[MgNTP]}{1 + K_1\,[Mg] + K_2[MgNTP]} \quad (Eq. 8)$$

$$V_{deg} = (k_{Ac}[H]^{n_{ac}} + k_{ba}[OH]^{n_{ba}} + k_{Mg}[Mg]^{n_{Mg}})\ [RNA]^{n_{RNA}} \quad (Eq. 9)$$

$$V_{precip} = \begin{cases} k_{precip}\ ([Mg_2PPi] - [Mg_2PPi]_{eq}) & if [Mg_2PPi] > [Mg_2PPi]_{eq} \\ 0 & if\ [Mg_2PPi] \leq [Mg_2PPi]_{eq} \end{cases} \quad (Eq. 10)$$

$$[Mg]_{tot} = [Mg] + [MgNTP] + 2 * [Mg_2NTP] + [MgHNTP] +]MgPPi] + 2 * [Mg_2PPi] + [MgHPPi]; (M1)$$

$$[NTP]_{tot} = [NTP] + [MgNTP] + [Mg_2NTP] + [MgHNTP] + [HNTP]; (M2)$$

$$[H]_{tot} = [H] + [MgHNTP] + [HNTP] + [HPPi] + 2 * [H_2PPi] + [MgHPPi] + [HHEPES]\ ; (M3)$$

$$[PPi]_{tot} = [PPi] + [MgPPi] + [Mg_2PPi] + [HPPi] + [H_2PPi] + [MgHPPi]; (M4)$$

$$[HEPES]_{tot} = [HEPES] + [HHEPES]\ ; (M5)$$

$$[H][NTP] = K_{eq,0}[HNTP] \quad (E1)\ ;\ [Mg][NTP] = K_{eq,1}[MgNTP] \quad (E2)$$

$$[Mg][MgNTP] = K_{eq,2}[Mg_2NTP] \quad (E3)\ ;\ [Mg][HNTP] = K_{eq,3}[MgHNTP] \quad (E4)$$

$$[Mg][PPi] = K_{eq,4}[MgPPi] \quad (E5)\ ;\ [Mg][MgPPi] = K_{eq,5}[Mg_2PPi] \quad (E6)$$

$$[H][PPi] = K_{eq,6}[HPPi] \ (E7) \ ; \ [H][HPPi] = K_{eq,7}[H_2PPi] \ (E8)$$

$$[Mg][HPPi] = K_{eq,8}[MgHPPi] \ (E9) \ ; \ [H][HEPES] = K_{eq,9}[HHEPES]; (E10)$$

<u>Nominal Initial conditions:</u>

$[Mg]_{tot}$=0.085 M

$[NTP]_{tot}$= 0.04M

$[H]_{tot}$ = 0.02 M (or if possible the equivalent of pH = 7.5)

$[PPi]_{tot}$ = 1e-18 M (for numerical stability reasons)

$[HEPES]_{tot}$= 0.04 M

$[RNA]_{tot} = 0$

$[T7RNAP]_{tot}$= 1e-7 M

To convert the 10 kb RNA concentration from mol/L to g/L, multiply by molecular weight of 5 million g/mol.

## Nomenclature:

| Parameter name | Symbol | Units | Value |
|---|---|---|---|
| **Total component concentrations of magnesium, nucleotide, proton, pyrophosphate, HEPES, RNA** | $[Mg]_{tot}$, $[NTP]_{tot}$, $[H]_{tot}$, $[PPi]_{tot}$, $[HEPES]_{tot}$, $[RNA]_{tot}$ | mol/L | Given as initial condition, then modelled [cf. code].<br>Nominal Initial conditions:<br>$[Mg]_{tot}$=0.085 M , $[NTP]_{tot}$= 0.04M<br>$[H]_{tot}$ = 0.02 M (or if possible the equivalent of pH = 7.5)<br>$[PPi]_{tot}$ = 1e-18 M (for numerical stability reasons)<br>$[HEPES]_{tot}$= 0.04 M, $[RNA]_{tot} = 0$<br>$[T7RNAP]_{tot}$= 1e-7 M |

| | | | |
|---|---|---|---|
| **$Mg^{2+}$, $NTP^{4-}$, $H^{+}$, $HEPES^{-}$ $PPi^{4-}$ $HNTP^{3-}$, $MgNTP^{2-}$, $Mg_2NTP$, $MgHNTP^{-}$, $MgPPi^{2+}$, $Mg_2PPi$, $HPpi^{3-}$, $H_2PPi^{2-}$, $MgHPPi^{-}$ and HHEPES free solution concentrations** | $[Mg]$, $[MgNTP]$, $[Mg_2NTP]$, $[MgHNTP]$, $[NTP]$, $[HNTP]$, $[PPi]$, $[H]$, $[MgPPi]$, $[Mg_2PPi]$, $[HPPi]$, $[H_2PPi]$, $[MgHPPi]$, $[HEPES]$, $[HHEPES]$ | mol/L | modelled |
| **T7RNAP enzyme concentration** | $[T7RNAP]_{tot}$ | mol/L | Given as initial condition, then modelled |
| **Dissociation equilibrium constants** | $K_{eq,0}$, $K_{eq,1}$, $K_{eq,2}$, $K_{eq,3}$, $K_{eq,4}$, $K_{eq,5}$, $K_{eq,6}$, $K_{eq,7}$, $K_{eq,8}$, $K_{eq,9}$ | mol/L | $10^{-6.95}$, $10^{-4.42}$, $10^{-1.69}$, $10^{-1.49}$, $10^{-5.42}$, $10^{-2.33}$, $10^{-8.94}$, $10^{-6.13}$, $10^{-3.05}$, $10^{-7.5}$ |
| **Transcription rate** | $V_{tr}$ | $\frac{mol}{L.hr}$ | modelled |
| **Degradation rate** | $V_{deg}$ | $\frac{mol}{L.hr}$ | modelled |
| **Precipitation rate** | $V_{precip}$ | $\frac{mol}{L.hr}$ | modelled |
| Transcription rate constant | $k_{app}$ | $\frac{L^2}{mol.U.hr}$ | Fitted: |
| Mg saturation constant | $K_1$ | $\frac{L^2}{mol}$ | Fitted: |
| MgNTP saturation constant | $K_2$ | $\frac{L^2}{mol.hr}$ | Fitted: |
| Acidic degradation rate constant | $k_{ac}$ | $\frac{L^2}{mol.hr}$ | Fitted: |
| Basic degradation rate constant | $k_{ba}$ | $\frac{L^2}{mol.hr}$ | Fitted: |
| Magnesium degradation rate constant | $k_{Mg}$ | $\frac{L^2}{mol.hr}$ | Fitted: |
| Reaction order with respect to RNA, proton, hydroxy and magnesium concentration | $n_{RNA}$, $n_{ac}$, $n_{ba}$, $n_{Mg}$ | / | Fixed to 1 |

| | | | |
|---|---|---|---|
| Precipitation rate constant | $k_{precip}$ | $hr^{-1}$ | Fixed to 0 |
| Equilibrium $Mg_2PPi$ concentration | $[Mg_2PPi]_{eq}$ | mol/L | 1.4 x $10^{-5}$ |
| RNA chain length | $N_{all}$ | / | 10,000 |

## Supplementary References:


1. CMC-Vaccines Working Group. A-Vax: applying quality by design to vaccines [Internet]. Bethesda, MD, USA: Parenteral Drug Association (PDA); 2012. Available from: https://www.dcvmn.org/IMG/pdf/a-vax-applying-qbd-to-vaccines_2012.pdf

2. TriLink BioTechnologies. CleanCap Technology: Leading the way in mRNA™ [Internet]. 2021 [cited 2021 Jan 12]. Available from: https://www.trilinkbiotech.com/cleancap

3. TriLink BioTechnologies. CleanCap Reagent AG for Co-transcriptional Capping of mRNA [Internet]. San Diego, CA, USA; 2020. Available from: https://www.trilinkbiotech.com/media/productattach/c/l/cleancapag_n7113_productinsert.pdf

4. TriLink BioTechnologies. CleanCap Reagent AU for Self-Amplifying mRNA [Internet]. San Diego, CA, USA; 2020. Available from: https://www.trilinkbiotech.com/media/productattach/c/l/cleancapau_n7114_productinsert.pdf

5. McNaught AD, Wilkinson A. IUPAC. Compendium of Chemical Terminology, 2nd ed. (the "Gold Book") [Internet]. IUPAC Compendium of Chemical Terminology. Oxford, UK: Blackwell Scientific Publications; 1997. Available from: https://goldbook.iupac.org/terms/view/A00543

6. Sobol' IM. On sensitivity estimation for nonlinear mathematical models. Mat Model [Internet]. 1990;2(1):112–118. Available from: http://www.mathnet.ru/php/archive.phtml?wshow=paper&jrnid=mm&paperid=2320&option_lang=eng

7. Sobol' IM. Sensitivity Estimates for Nonlinear Mathematical Models. Mathematical Modeling and Computational experiment. 1993.

8. Sobol' IM, Asotsky D, Kreinin A, Kucherenko S. Construction and Comparison of High-Dimensional Sobol' Generators. Wilmott [Internet]. John Wiley & Sons, Ltd; 2011 Nov 1;2011(56):64–79. Available from: https://doi.org/10.1002/wilm.10056

9. Bratley P, Fox BL. Algorithm 659: Implementing Sobol's Quasirandom Sequence Generator. ACM Trans Math Softw [Internet]. New York, NY, USA: Association for Computing Machinery; 1988 Mar;14(1):88–100. Available from: https://doi.org/10.1145/42288.214372

10. Kucherenko S. SobolHDMR: a general-purpose modeling software. Methods Mol Biol. United States; 2013;1073:191–224.

11. Bancel S, Issa, William J, Aunins, John G, Chakraborty T. Manufacturing methods for production of RNA transcripts [Internet]. USA: United States Patent and Trademark Office; WO/2014/152027; PCT/US2014/026835; US20160024547A1, 2014. Available from: https://patentimages.storage.googleapis.com/7a/bb/8f/5ce58cdaa18a0d/US20160024547A1.pdf (accessed on 10.Nov.2020)

12. Scorza Francesco Berlanda, Yingxia Wen, Andrew Geall, Frederick Porter. RNA purification methods [Internet]. 20160024139, EP2970948A1; WO2014140211A1, 2016 [cited 2018 May 1]. Available from: https://patents.google.com/patent/EP2970948A1/no

13. Heartlein M, Derosa F, Dias A, Karve S. Methods for purification of messenger rna [Internet]. USA; DK14714150.1T; PCT/US2014/028441, 2014. Available from: https://patents.google.com/patent/DK2970955T3/en (accessed on 15.Dec.2019)

14. Funkner A, Dorner S, Sewing S, Kamm J, Broghammer N, Ketterer T, Mutzke T. A method for producing and purifying rna, comprising at least one step of tangential flow filtration [Internet]. Germany: World Intellectual Property Organization; PCT/EP2016/062152; WO/2016/193206, 2016. Available from: https://patentscope.wipo.int/search/en/detail.jsf?docId=WO2016193206 (accessed on 10.Oct.2020)

15. Kis Z, Shattock R, Shah N, Kontoravdi C. Emerging Technologies for Low-Cost, Rapid Vaccine Manufacture. Biotechnol J [Internet]. John Wiley & Sons, Ltd; 2019 Jan 1;14(1):1800376. Available from: https://doi.org/10.1002/biot.201800376

16. Kis Z, Kontoravdi C, Dey AK, Shattock R, Shah N. Rapid development and deployment of high-volume vaccines for pandemic response. J Adv Manuf Process [Internet]. 2020/06/29. John Wiley & Sons, Inc.; 2020 Jul;2(3):e10060. Available from: https://www.ncbi.nlm.nih.gov/pmc/articles/PMC7361221/

17. Centre for Process Innovation Limited. Telephone and email correspondence with biopharmaceutical manufacturing experts from the Centre for Process Innovation Limited, UK - Jul 2018. Darlington, UK: CPI; 2020.

18. Kis Z, Kontoravdi C, Shattock R, Shah N. Resources, Production Scales and Time Required for Producing RNA Vaccines for the Global Pandemic Demand [Internet]. Vaccines. 2021. Available from: https://www.mdpi.com/2076-393X/9/1/3/htm
